# Latent thermodynamic flows: unified representation learning and generative modeling of temperature-dependent behaviors from limited data

**DOI:** 10.1039/d5sc06402c

**Published:** 2026-01-02

**Authors:** Yunrui Qiu, Richard John, Lukas Herron, Pratyush Tiwary

**Affiliations:** a Institute for Physical Science and Technology, University of Maryland College Park MD 20742 USA ptiwary@umd.edu; b Institute for Health Computing, University of Maryland Bethesda MD 20852 USA; c Department of Physics, University of Maryland College Park MD 20742 USA; d Biophysics Program, University of Maryland College Park MD 20742 USA; e Department of Chemistry and Biochemistry, University of Maryland College Park MD 20742 USA

## Abstract

Accurate characterization of equilibrium distributions in complex molecular systems, and their dependence on environmental factors such as temperature, is crucial for understanding thermodynamic properties and transition mechanisms. However, obtaining converged sampling of these high-dimensional distributions using approaches like molecular dynamics simulations often incurs prohibitive computational costs. And the absence of informative low-dimensional representations for these distributions hampers interpretability and many downstream analyses. Recent advances in generative AI, particularly flow-based models, show promise for efficiently modeling molecular equilibrium distributions; yet, without tailored representation learning, their generative performance on high-dimensional distributions remains limited and inexplicable. In this work, we present Latent Thermodynamic Flows (LaTF), an end-to-end framework that seamlessly integrates representation learning with generative modeling. LaTF unifies the State Predictive Information Bottleneck with Normalizing Flows to simultaneously learn low-dimensional representations, *i.e.*, collective variables, classify metastable states, and generate equilibrium distributions across temperatures beyond the training data. The joint optimization of representation learning and generative modeling allows LaTF to mutually enhance both components, making optimal use of costly simulation data to accurately reproduce the system's equilibrium behaviors over the meaningful latent representation that captures its slow, essential degrees of freedom. We demonstrate LaTF's effectiveness across diverse systems, including a model potential, the Chignolin protein, and a cluster of Lennard-Jones particles, with thorough evaluations and benchmarking using multiple metrics and extensive simulations. Moreover, we apply LaTF to a RNA tetraloop system, where despite using simulation data from only two temperatures, LaTF reconstructs the temperature-dependent structural ensemble and melting behavior, consistent with experimental and prior extensive computational results.

## Abstract 2

Unified learning of low-dimensional representations and generative modeling of free energy surfaces for complex molecular systems mutually reinforce each other, allowing reliable prediction of temperature-dependent behavior from limited data.

## Graphical abstract

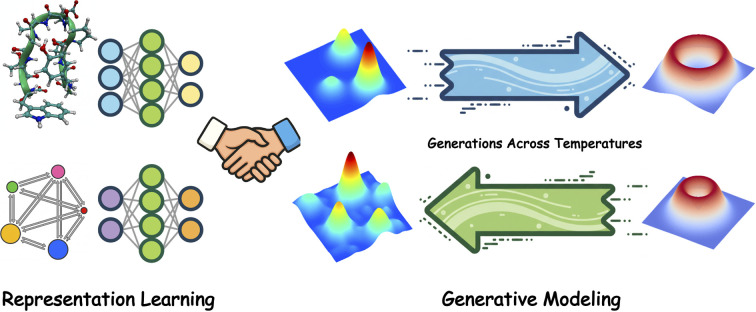

## 1 Introduction

Quantifying microscopic molecular equilibrium distributions and deriving their macroscopic properties is a ubiquitous task across physics, chemistry, materials science, and biology. For example, predicting the populations of protein conformations is critical for elucidating their functional mechanisms and the design of therapeutic molecules.^1–6^ Similarly, determination of relative stability for material phases over various thermodynamic conditions is essential for constructing phase diagrams and guiding new material designs.^7–9^ Statistical mechanics provides a rigorous framework for computing microscopic equilibrium distributions under given environmental constraints, from which all thermodynamic observables of interest can then be calculated.

Molecular systems of practical interest possess extremely high-dimensional configurational spaces, making it difficult to sample and express their global equilibrium distributions using traditional tools such as Molecular Dynamics (MD) simulations and standard density estimators. These challenges are further exacerbated when studying environment-dependent behaviors, where simulations and estimations must be repeated for each condition. Even when high-dimensional distributions are successfully obtained, they remain difficult to analyze and interpret. Therefore, evaluating the equilibrium distribution on a set of low-dimensional, physically meaningful collective variables (CVs) is a more tractable strategy. This not only enables reliable quantification of thermodynamic properties but also facilitates understanding of the molecular mechanisms underlying complex dynamical processes.^10–14^ Over the past decades, numerous enhanced sampling techniques, such as umbrella sampling^10^ and metadynamics,^12^ have been developed to sketch the free energy surface (FES) on carefully chosen CVs that capture slow transitions between long-lived metastable states.^15–17^

Recently, the integration of machine learning with physical principles has driven the development of many methods for CV identification and equilibrium distribution prediction. For example, the Information Bottleneck (IB) principle^18^ and the Variational Approach for Markov Processes^19^ have inspired numerous methods for CV construction.^20–24^ Several active subfields have emerged from these frameworks, where new methods continue to be developed and applied across chemical, material, and biological systems.^25–35^ In parallel, deep generative models have attracted growing interest for generating equilibrium structural ensembles under different environmental conditions. For instance, Boltzmann generators^36^ and their methodological variants^37–41^ have been developed to generate unbiased samples (at varying temperatures) by constructing invertible flow-based mappings between equilibrium distributions and Gaussian priors. Similarly, thermodynamic maps^42,43^ and their extensions^44–46^ are capable of inferring how equilibrium distributions vary with thermal variables, including temperature and pressure, using diffusion models trained on limited simulation data. Despite these advances, most generative models struggle to generate high-fidelity atomic structures due to the high dimensionality of the task and the cost of obtaining sufficient equilibrium training data. Typically, the generation performance is only evaluated by projecting the generated samples onto a few selected CVs.^36–43^ However, so far, CV construction and distribution modeling have been treated as two separate tasks and addressed independently.

In the realm of CV construction, most efforts prioritize capturing the slowest dynamical transitions between metastable states, often overlooking their potential for generative tasks. Conversely, generative models emphasize improving sampling fidelity, while giving little attention to the benefits that could arise from learning meaningful representations. Yet, they inherently face the curse of dimensionality: theory validates that generation error grows exponentially with input dimensionality,^47,48^ and experiments show that achieving accurate, generalizable generation demands increasingly large datasets as dimensionality rises.^48,49^ This compartmentalized development mirrors similar trends in the broader generative AI field.^50–53^ Consequently, uniting representation learning and generative modeling holds both theoretical and practical significance. Projecting data onto a low-dimensional manifold enables the training of more expressive, faithful and explainable generative models with much smaller neural network architectures, and accurate modeling of the projected distributions could guide the refinement of these projections.

**Fig. 1:**
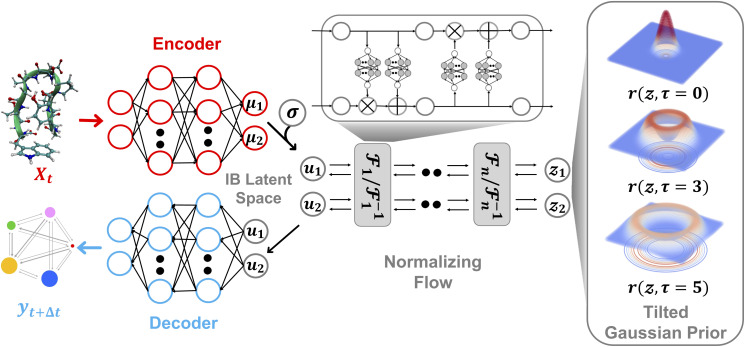
Architecture of the Latent Thermodynamic Flows (LaTF) model. The LaTF model consists of three components which are jointly trained together: an encoder that projects molecular descriptors ***X***_*t*_ at time *t* into a 2D Information Bottleneck latent space {*µ*_*i*_}_*i*=1_^2^; a normalizing flow employing real-valued non-volume preserving transformations 
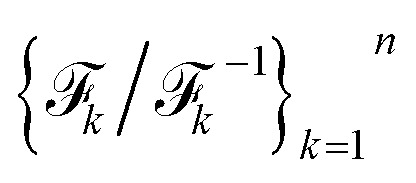
 to establish a reversible mapping between the encoded Gaussian distribution 
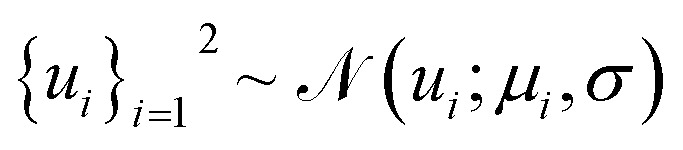
 and the exponentially tilted Gaussian prior *r*(***z***, *τ*); and a decoder that predicts metastable state labels ***y***_*t*+Δ*t*_, representing the state into which the input configuration transits after a lag time Δ*t*. The right most column visualizes the 2D tilted Gaussian prior distributions for several values of the tilting parameter *τ*, with *τ* = 0 representing the standard Gaussian distribution.

In this study, we present a unified framework that seamlessly combines representation learning with generative modeling. Our proposed approach, termed Latent Thermodynamic Flows (LaTF), integrates the strengths of two powerful models: State Predictive Information Bottleneck (SPIB)^24^ and Normalizing Flows (NFs)^54,55^ (see model architecture in Fig. 1). SPIB has demonstrated great effectiveness across diverse systems in extracting CVs that capture the slowest dynamical modes from high-dimensional molecular descriptors, while also partitioning configurations into metastable states.^25,26^ NFs are powerful deep generative models that have been applied to approximate complex molecular equilibrium distributions and free energy calculations.^36–39,56–58^ By employing the NF as a bijective transformation between the latent IB distribution and a prior distribution, we formulate a unified objective that enables the simultaneous training of both SPIB and the NF. We show that the joint training scheme offers benefits complementary to SPIB and NF: it facilitates the optimization of the encoder and decoder, leading to improved delineation of metastable state boundaries, and enables explicit quantification and accurate sampling of the stationary distribution over physically meaningful CVs.

Additionally, in place of the conventional standard Gaussian prior, we employ an exponentially tilted Gaussian distribution for the NF, which expands the volume of high-density regions to facilitate metastable state separation and enable more physically realistic interpolation of transition pathways. By further introducing a temperature-steerable parameter into the tilted prior distribution, LaTF reliably captures nontrivial variations in the IB FES across a broad temperature range, even when trained on data from only a few temperatures. We validate the broad applicability of LaTF across three diverse systems: the Chignolin protein, a cluster of Lennard-Jones particles, and an RNA GCAA tetraloop. In each case, we benchmark our results against extensive computational results or previous experimental studies. Notably, LaTF predicts the temperature-dependent FES and melting temperature of the RNA tetraloop using simulation data collected at only two temperatures, showing agreement with established references. Therefore, we expect that LaTF will serve as a versatile framework for complex systems, providing robust end-to-end capabilities for CV extraction, metastable state identification, pathway interpolation, equilibrium distribution estimation, and temperature-dependent thermodynamic property prediction.

## 2 Results

### 2.1 Setting up latent thermodynamic flows (LaTF): unifying representation and generation with tilted Gaussian prior

A schematic illustration of the LaTF model architecture is shown in Fig. 1. The LaTF model inherits its ability to identify meaningful CVs and metastable states for complex molecular systems from the well-established SPIB model. SPIB employs a variational autoencoder-like architecture that encodes high-dimensional molecular descriptor ***X***_*t*_ into a low-dimensional IB space ***z*** and decodes it to predict the metastable state ***y***_*t*+Δ*t*_ into which the input configuration transits after a lag time Δ*t*. In line with the IB principle,^18^ SPIB adopts the following loss function:1
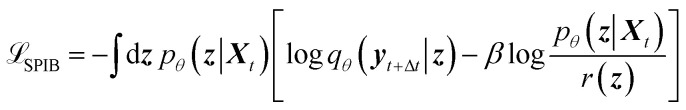
where *p*_*θ*_(***z***|***X***_*t*_) denotes the posterior distribution, which could be estimated using a Gaussian encoder that maps the input data into the IB space, *q*_*θ*_(***y***_*t*+Δ*t*_|***z***) represents the decoder-predicted probability of the future states (*θ* denotes neural network parameters), and *r*(***z***) is the prior distribution over IB space, defined as a modified variational mixture of posteriors prior (VampPrior). The loss function includes weight *β*, a tunable parameter that balances future-state prediction accuracy with regularization of the encoded posterior towards Gaussian mixture prior. Once trained, the learned IB space ideally captures slow inter-state transitions with timescales longer than Δ*t* and enables clear separation of metastable states. SPIB is trained through an iterative and self-consistent scheme where the short-lived states are merged into long-lived ones and input configurations are relabeled on-the-fly with their most probable future-state labels to maximize state metastability^32^ (see SI for more SPIB details).

Mapping the FES over SPIB-derived CVs with enhanced sampling has proven to be broadly applicable and meaningful across diverse systems, including drug binding and unbinding,^25^ biomolecular conformational changes,^32^ and crystal polymorph nucleation.^26^ While the analytical multi-modal VampPrior used in SPIB provides better regularization than a single Gaussian prior, it remains limited in generative tasks. Here, we overcome the limitation by replacing it with more expressive generative model, namely the NF. As shown in Fig. 1, the NF consists of a sequence of bijective transformations that serve as a change of variables, mapping the encoded IB distribution to an easily sampled prior distribution. We utilize the real-valued non-volume preserving (RealNVP) transformation,^55^ which offers an explicitly computable Jacobian determinant (more details in Methods). Inspired by previous studies,^50–53,59^ modeling the posterior distribution *p*_*θ*_(***z***|***X***_*t*_) with NF mapping 
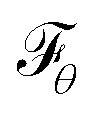
 could be mathematically expressed as an integral involving the Dirac delta function:2
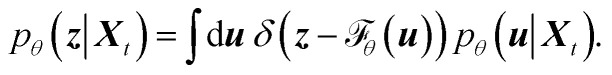


Here, we introduce a new random variable ***u***, which obeys a Gaussian distribution conditioned on the encoded representation, *i.e.*, 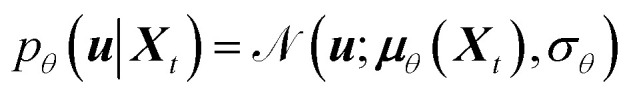, and is flowed to obtain the posterior distribution (Fig. 1). The variance *σ*_*θ*_ is treated as a learnable, input-independent parameter. Unlike SPIB, which directly regularizes the encoded distribution to a multi-modal Gaussian in a brute-force manner, LaTF adopts a two-step transformation, *e.g.*, encoding followed by flow refinement, allowing for a more expressive representation of the encoded distribution and enabling a better alignment between the posterior and the prior. The unified loss function for jointly training all components in LaTF could be derived by integrating eqn (1) and (2) (see more derivation details in the SI):3
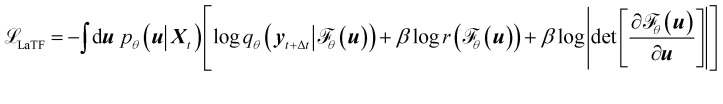


While the first term captures the reconstruction error associated with future-state prediction, the second and third terms jointly regularize the encoder and NF to ensure accurate alignment between the posterior and prior distributions. This unified objective enables the model to extract structural features that are highly informative for inferring transition dynamics over lag time Δ*t*, while simultaneously providing an explicit and reliable density estimator over these representations. Additionally, the incorporation of the NF also supports the utilization of a more generalized and flexible prior, for which we adopt an exponentially tilted Gaussian distribution^60^ (see examples in Fig. 1):4
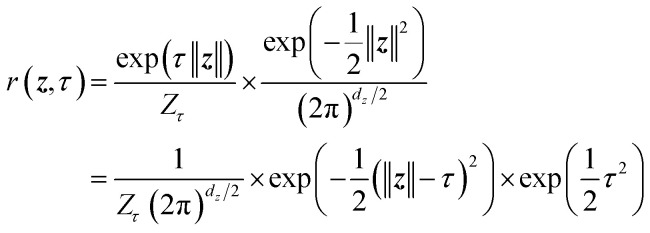
where *τ* is the tilting factor, *d*_*z*_ is IB space dimensionality, and *Z*_*τ*_ denotes the analytically tractable normalization constant (see Methods for details). Exponential tilting is a widely used procedure in diverse fields such as statistical mechanics, large deviations, and importance sampling, where it reweights the original distribution so that low-likelihood configurations become more typical under the tilted measure. The standard Gaussian concentrates its maximum probability at a single point, which will compress samples from complex distributions into a small region and thereby make it difficult for NF models to generate different multi-modal samples from nearby prior locations. By contrast, the tilted Gaussian is radially symmetric and attains its maximum probability on the hyperspherical shell defined by ‖***z***‖ = *τ*, providing a much larger volume for the high-density region, facilitating better accommodation and separation of complex multi-modal data. As we will show later, the tilted Gaussian also offers substantial advantages in predicting temperature-dependence of equilibrium distributions.

So far, two parameters are particularly crucial for the LaTF model: the IB latent dimension *d*_*z*_ and the tilting factor *τ*. Throughout this study, we adopt a two-dimensional IB space to maintain a balance between effective dynamical representation, interpretability, and generative accuracy. However, the IB dimensionality could be extended when needed to capture richer and more detailed kinetic information. We justify our choice by benchmarking against multi-dimensional CVs obtained from time-lagged independent component analysis (tICA)^61,62^ and against metastable states identified through Markov state modeling. For both the Chignolin protein and RNA tetraloop folding systems, we find that the two-dimensional IB representation successfully captures the essential dynamical information embedded in the multi-dimensional tICA space, while also clearly separating the relevant metastable states, consistent with prior studies.^32,63^ Validation details and further discussion regarding the choice of IB dimensionality are provided in the SI. Moreover, we select the optimal tilting factor *τ* using the Kullback–Leibler (KL) divergence between the generated and reference distributions from simulation data. The value of *τ* that yields the lowest generation divergence will be adopted (more details for LaTF training are presented in Methods).

### 2.2 Benchmarking LaTF for model potential and Chignolin in explicit water

**Fig. 2:**
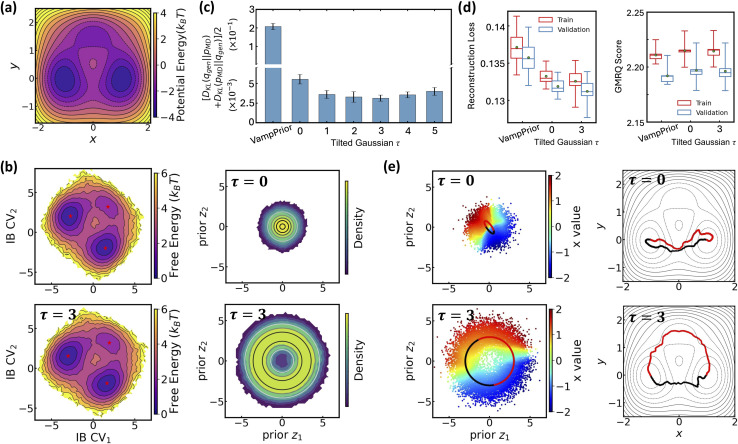
Benchmarking LaTF performance on 2D three-hole potential system at temperature *k*_B_*T* = 1. (a) Analytical potential energy surface. (b) Projections of simulation data onto the IB latent spaces (left) and prior spaces (right) by LaTF models trained with different tilting factors. Asterisks indicate the configurations with the highest likelihood quantified by LaTF in each state. Contour lines of the analytical prior illustrate LaTF's ability to learn an effective mapping between IB latent and prior distributions. (c) Symmetric KL divergence between the reference IB distribution (from the validation dataset) and the distributions generated by vanilla SPIB with VampPrior and LaTF models with different tilting factors. (d) Comparison of the quality of metastable state assignment between vanilla SPIB and LaTF. All uncertainties in (c) and (d) are derived *via* five-fold cross-validation. (e) Interpolation of transition pathways in the prior spaces (left), where background shows projected data colored by their *x*-coordinate values. Interpolated samples are mapped back to *x* − *y* coordinate space *via* nearest neighbors in the IB space (right).

The effectiveness of LaTF and the exponentially tilted Gaussian prior is demonstrated on both a model potential and the well-studied Chignolin protein system, as we have accurate results to compare with. The 2D potential shown in Fig. 2(a) features two deep basins connected by two reaction channels, with the upper channel containing a local minimum. Langevin dynamics simulation of a single particle on this potential surface is performed and used to train LaTF models with varying tilting factors (see Methods for simulation and training details).

Visual inspection (Fig. 2(b) and S2) reveals that, after appropriate rotation, the IB space learned by LaTF closely resembles the original potential surface, and the flow-transformed distribution in prior space aligns well with the analytical prior, indicating that LaTF correctly learns meaningful IB space and meanwhile accurately captures its distribution. To quantitatively evaluate the performance, we apply three metrics and compare LaTF with a vanilla SPIB model (with VampPrior) trained under identical settings, except without the NF module. The KL divergence between IB distributions derived from generation and simulation data (Fig. 2(c)) confirms that incorporating flexible NF model largely improves alignment between the encoded IB distribution and the prior, with the tilted Gaussian prior providing further improvements in fidelity. Introducing the NF and tilted Gaussian prior also reduces LaTF's reconstruction loss for future-state prediction (*i.e.*, the first term in eqn (3)) compared to the vanilla SPIB, suggesting improved modeling of inter-state transitions and better metastable state classification (Fig. 2(d), left). This is further supported by the improved performance of the resulting Markov State Model (MSM)^64^ constructed using state labels derived from the decoder, as assessed by the generalized matrix Rayleigh quotient (GMRQ) score^65^ (Fig. 2(d), right). The GMRQ quantifies state metastability and the model's capacity to capture slow dynamics, with higher scores indicating better performance (see Methods for details). These results demonstrate that the unified training framework promotes mutual enhancement between representation learning and generative modeling, with the encoder, decoder, and NF components performing better when trained together.

Additionally, the flexibility of the tilted Gaussian enables richer structure in the prior space, which in turn supports more physically realistic interpolations in the IB space. Since the *x*-coordinate is a good proxy for the committor function in this system, we color the data flowed to the prior space by their *x*-values (Fig. 2(e), left column). The standard Gaussian, which is the most common prior used in generative models to approximate molecular equilibrium distributions, collapses the data into a narrow high-density region, whereas the tilted Gaussian distributes them more uniformly, clearly distinguishing the two reaction channels. Using the trained NF model and associated Jacobian determinant to evaluate configuration likelihoods, we identify the most probable states in each basin and interpolate pathways between them. For the standard Gaussian prior we use spherical linear interpolation,^66^ while for the tilted Gaussian prior, taking advantage of its structured geometry, we directly apply simplest linear interpolation between the central angles and radii of the path endpoints (see SI for details). The standard Gaussian prior produces pathways confined to a single reaction channel, whereas the tilted Gaussian recovers transition pathways across two distinct channels (Fig. 2(e), right column).

**Fig. 3:**
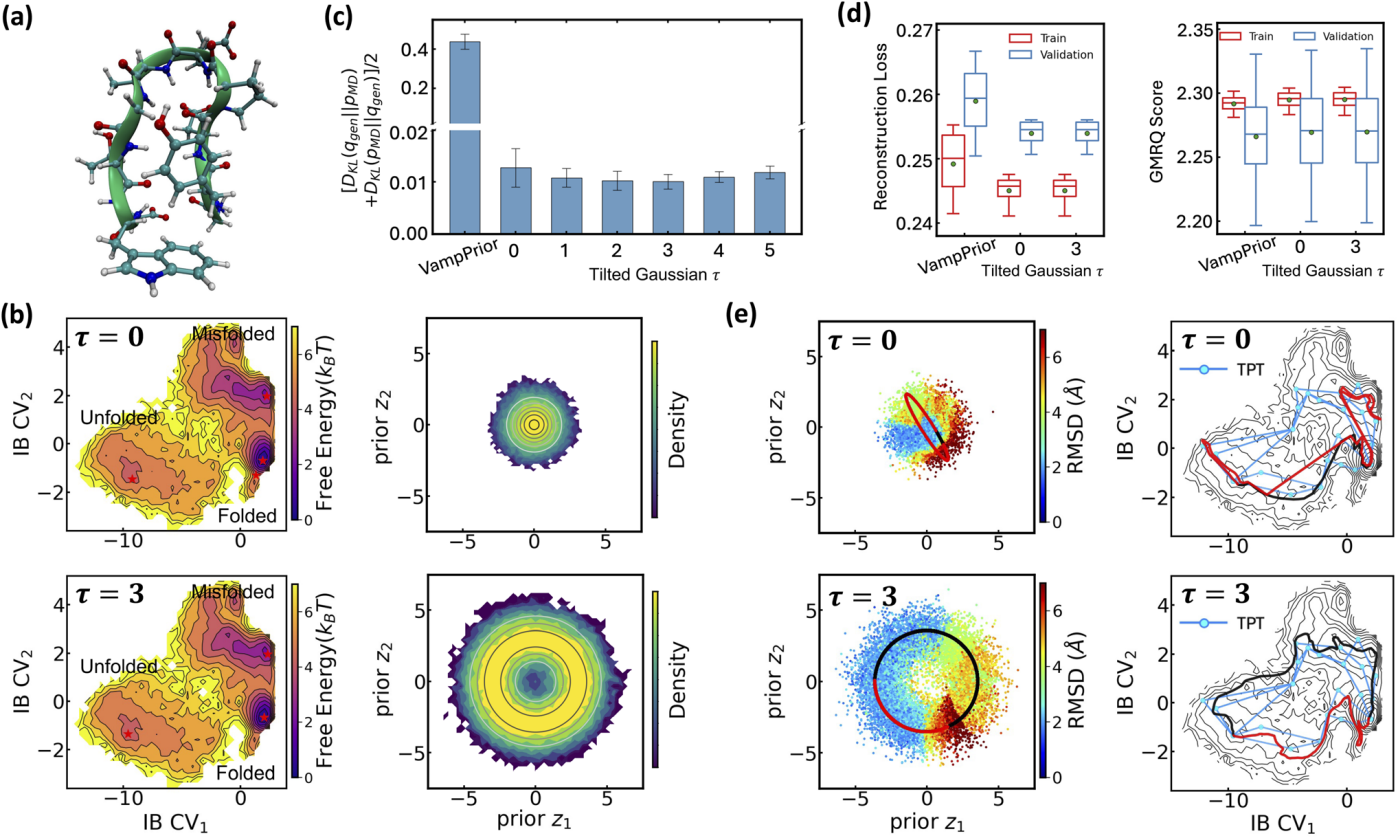
Benchmarking LaTF performance on Chignolin protein system at temperature 340 K. (a) PDB structure of the Chignolin protein (PDB: 1UAO). (b) Projections of simulation data onto the IB latent space (left) and prior space (right) by LaTF models trained with different tilting factors. Asterisks indicate the configurations with the highest likelihood quantified by LaTF in each state. Contour lines of the analytical prior illustrate LaTF's ability to learn an effective mapping between latent and prior distributions. (c) Symmetric KL divergence between the reference IB distribution (from the validation dataset) and the distributions generated by vanilla SPIB with VampPrior and LaTF with different tilting factors. (d) Comparison of the quality of metastable state assignment between vanilla SPIB and LaTF. All uncertainties in (c) and (d) are derived *via* five-fold cross-validation. (e) Interpolation of transition pathways in the prior space (left), where the background shows projected data colored by the heavy-atom RMSD relative to the PDB structure. The interpolated pathways are flowed to the IB space and compared with the top five highest-flux pathways identified by Transition Path Theory (right).

Extending the above analysis, we further demonstrate LaTF's robustness on a ten-residue protein system, Chignolin (PDB: 1UAO; Fig. 3(a)),^67^ using a long unbiased MD trajectory (∼35 µs) simulated at 340 K in all-atom resolution including explicit water (see Methods for simulation and LaTF training details). Consistent with earlier results, the LaTF model clearly distinguishes Chingolin's folded, unfolded, and misfolded states in the IB latent space (Fig. 3(b)). The close agreement between the generated and reference FES in the IB space (Fig. S3) underscores LaTF's advantage in optimally leveraging data to accurately model distributions over physically meaningful variables. Meanwhile benefiting from the NF model, which flexibly refines the mismatch between posterior and prior, LaTF outperforms vanilla SPIB in IB distribution approximation, future-state prediction, and metastable state classification (Fig. 3(c and d)). Furthermore, analyses of the implied time scales and the Chapman–Kolmogorov test provide additional validation of LaTF's superior performance over SPIB (see Fig. S4 and SI for details).

Notably, the advantages of the tilted Gaussian prior are further emphasized when performing pathway interpolation for the Chignolin protein. As shown in Fig. 3(e), visualizing the distribution of heavy-atom root-mean-square deviation (RMSD) relative to the PDB structure in prior space reveals the limitation of the standard Gaussian: it places folded structures in a high-density region, while misfolded and unfolded structures lie at opposite low-density boundaries, connected only by a narrow region that, although critical for folding transitions, is poorly represented. In contrast, the tilted Gaussian spreads the configurations more uniformly, clearly resolving folded and unfolded states and connecting them through two distinct and well-represented transition regions. Even with spherical linear interpolation, the standard Gaussian prior fails to produce pathways consistent with those identified by Transition Path Theory^68–70^ (TPT; see SI for more information), whereas simple linear interpolation on the tilted prior yields pathways that align with the TPT results. Further back-mapping of samples interpolated with the tilted prior, using nearest neighbors from simulation data (based on Euclidean distance in IB space), reveals two distinct folding mechanisms that differ in the sequence of H-bond breaking involving residue Asp3, Thr6, and Thr8 (see Fig. S5), consistent with findings from previous studies.^71,72^ Additionally, we evaluate the folding committor distribution across the IB space and identify two saddle points corresponding to distinct folding pathways on the free-energy landscape. These saddle points lie within regions where the committor is approximately 0.5, further demonstrating the ability of the IB representation to separate metastable states and capture kinetically meaningful intermediates (see Fig. S14 and SI for more details).

Results from both systems confirm the effectiveness of LaTF, which we attribute to its conjugate training framework and structured prior. Many existing approaches employ NFs for full-coordinate structure generation, however, they are generally restricted to implicit-solvent systems, demand extensive preprocessing of molecular Cartesian coordinates and exhibit limited performance in data-sparse yet critical transition regions.^36,38,39^ Differently, LaTF serves as a post-analysis framework that efficiently uses explicit-solvent simulation data to model equilibrium distributions over a representative latent space, which largely reduces generative uncertainty and provides an effective distance metric for backmapping to full-coordinate structures. In the next section, we further evaluate LaTF's data efficiency and physical transferability across temperatures.

### 2.3 Quantifying LaTF's ability to enable sampling at out-of-distribution temperatures

#### 2.3.1 Temperature-steerable tilted Gaussian prior

Temperature is a fundamental thermodynamic variable that sets the scale of thermal fluctuations relative to the potential energy. Although the effect of temperature is conceptually simple, when considered in microscopic coordinates and invoking equipartition theorem, its effect on macroscopic thermal properties is highly non-trivial. As a result, the development of methods for predicting how thermodynamic properties respond to changes in temperature has become increasingly important in recent years. Existing generative models typically handle temperature-dependent inference by encoding temperature into the prior distribution (*e.g.*, *via* variance modulation of a Gaussian prior),^36,38^ or incorporating it explicitly as a model input,^74^ or employing a combination of both approaches.^38,43^ Here, we adopt the first strategy by introducing a temperature-steerable parameter into LaTF's tilted Gaussian prior to allow inference of temperature-dependent FES in IB space. Specifically, we define the temperature-steerable tilted Gaussian as:5
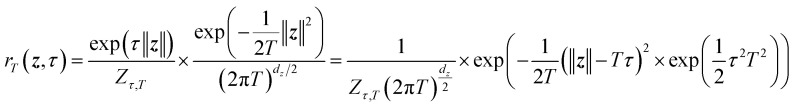
where *T* is the temperature-steerable parameter and *Z*_*τ*,*T*_ denotes normalization factor (more details in Methods). Completing the square for *r*_*T*_(***z***, *τ*) reveals that both its variance and the radius at which the probability is maximized scale proportionally with temperature. This construction enables the prior to better capture entropic contributions, which can shift and reshape free-energy basins relative to those defined solely by potential-energy minima. The proposed formulation thus enables accurate FES modeling while capturing temperature-specific variations. When *τ* = 0, the distribution reduces to a standard Gaussian whose variance scales linearly with temperature *T*, a form adopted in previous generative models.^36,38^ However, as we later show, this variance-only adjustment can be inadequate for inferring temperature-dependent FES. Since the low-temperature Gaussian is always superimposed within the high-temperature one, the higher-temperature generations may significantly be influenced by low-temperature distributions, which can introduce noticeable deviations when the FES changes significantly across temperatures.

#### 2.3.2 Inferring temperature-dependent behaviors of Chignolin protein

**Fig. 4:**
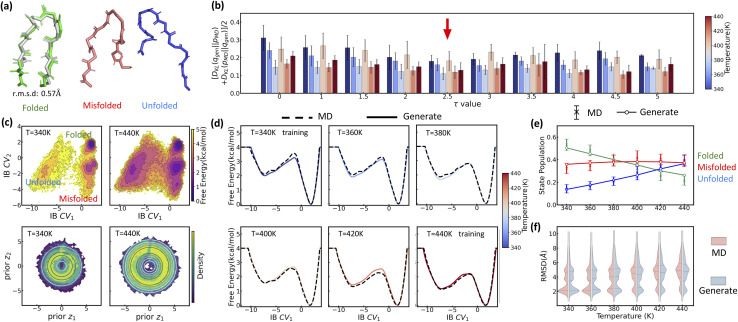
Evaluating LaTF performance in inferring temperature-dependent equilibrium distributions of Chignolin protein system using MD data at 340 K and 440 K. (a) Backbone structures of Chignolin with the highest likelihood in three metastable states (colored), shown alongside the PDB structure (gray) for comparison. (b) Symmetric KL divergence between LaTF-generated and simulation-derived IB distributions. For training temperatures (340 K & 440 K, shaded), divergence is computed on validation data; for other temperatures, it is quantified using the full MD data. Uncertainties are estimated from five-fold cross-validation. The optimal *τ* = 2.5 is chosen (red arrow) to minimize the divergence for only training temperatures. (c) FES constructed from encoded MD data in IB latent space, and density map of flow-transformed encoded data in the prior space for 340 K and 440 K. Analytical priors are shown in contour lines, and the density is normalized and estimated by the histogram approach. (d) Comparison of FES along IB CV_1_ from generated samples (solid) and long MD simulations (dashed) across six temperatures. (e) Populations of metastable states decoded from LaTF-generated samples across six temperatures; reference values and uncertainties are estimated *via* Bayesian MSMs.^73^ (f) Heavy-atom RMSD distributions relative to PDB structure for MD data and LaTF-generated samples, reconstructed to all-atom structures using nearest neighbors in latent space. Distribution quartiles are marked with dashed lines.

For the first benchmark study, we evaluate LaTF's performance on three tasks using the Chignolin protein system (Fig. 4(a)), comparing against extensive unbiased MD simulations conducted at six temperatures from 340 K to 440 K (see Methods for details). The first task for this benchmark system involves generating FES at intermediate temperatures using long simulation data from only the extremal temperatures, 340 K and 440 K. As before, the optimal *τ* is selected as 2.5 to minimize the KL divergence between generated and simulated distributions at the training temperatures (Fig. 4(b)). Visualizations of the FES in IB space and the associated density in prior space confirm that LaTF simultaneously identifies meaningful CVs and accurately captures the equilibrium distribution (Fig. 4(c)). The most likely structure in the folded state scored by LaTF aligns closely with the reference NMR structure (Fig. 4(a)). Notably, the temperature-steerable tilted Gaussian prior (*τ* > 0) consistently yields lower generation errors than the standard temperature-dependent Gaussian (*τ* = 0), with similar accuracies observed at both training and unseen temperatures (Fig. 4(b) and (d)). These results highlight LaTF's strong capability for generalizable ensemble generation at reduced cost. Meanwhile, LaTF's decoder can assign generated samples in IB space to metastable states, enabling inference of temperature-dependent state populations. These predictions agree well with those from MSMs built on long MD trajectories using the same state definitions (Fig. 4(e)). Finally, we reconstruct structural ensembles across temperatures by backmapping generated samples to their nearest neighbors in IB space encoded from training data (based on Euclidean distance). The resulting RMSD distributions closely match those from simulations (Fig. 4(f)), demonstrating LaTF's generative power of structural ensembles, which stems from both expressive CVs and a powerful latent-to-prior mapping.

The second task for the Chignolin system evaluates LaTF's performance under limited data availability. We train the LaTF model using only first 1 µs simulation segments from 340 K and 440 K, where the energy landscape is more thoroughly sampled at 440 K but the unfolded state is sparsely sampled at 340 K. As shown in Fig. S6, incorporating data from both temperatures into training significantly refine the estimation of the low-temperature FES, particularly in the transition and unfolded regions. Remarkably, despite the limited data, LaTF remains robust in extracting meaningful CVs and capturing both the FES topology and its temperature dependence.

We then proceed to the third task, in which LaTF is trained using long simulation data from two relatively high temperatures (*e.g.*, 380 K and 440 K) and used to generate FES at lower temperatures. As shown in Fig. S7, the results are consistent with previous findings and confirm that LaTF captures the correct temperature dependence, although the generation errors increase slightly at lower temperatures. Altogether, these three tasks demonstrate that LaTF with a tilted prior achieves strong physical transferability across temperatures, even under data-scarce conditions.

#### 2.3.3 Predicting temperature-driven transitions in the Lennard-Jones 7 cluster

**Fig. 5:**
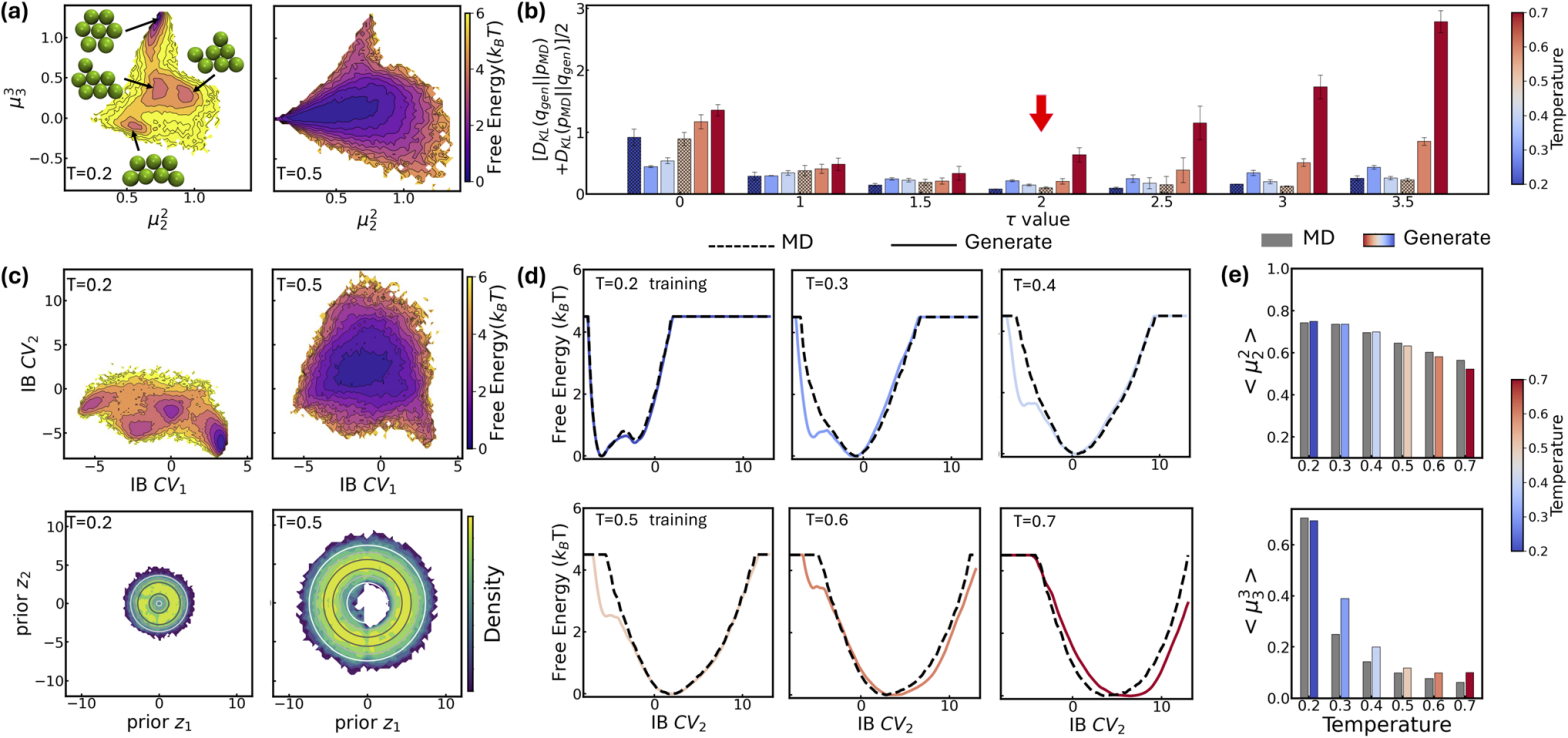
Inferring the temperature dependence of LJ7 system from data at 0.2*ε*/*k*_B_ and 0.5*ε*/*k*_B_*via* LaTF. (a) Projected FES onto the second and third moments of coordination numbers, *µ*_2_^2^ and *µ*_3_^3^, at 0.2*ε*/*k*_B_ and 0.5*ε*/*k*_B_. For each metastable state at 0.2*ε*/*k*_B_, the structure with the highest likelihood scored by LaTF is shown. (b) Symmetric KL divergence between distributions generated by LaTF and those from MD simulations. For training temperatures (0.2*ε*/*k*_B_ and 0.5*ε*/*k*_B_), the divergence is computed using the validation dataset; for other temperatures, the full MD data are used. The optimal *τ* = 2 is chosen (red arrow) to minimize the divergence for training temperatures (temperatures apart from 0.2*ε*/*k*_B_ and 0.5*ε*/*k*_B_ were not used for optimal *τ* calculation). Hatched bars indicated training temperatures. (c) FES in the IB space and density in the prior space estimated from encoded and flow-transformed MD data. Contour lines indicate the analytical tilted prior, and the density is normalized and estimated by the histogram approach. (d) Comparison of LaTF-generated FES (solid) with MD-derived FES (dashed) across temperatures. (e) Generative (colored) *vs.* reference (gray) means of the second and third moments of coordination numbers across temperatures. Generated samples are mapped to full-coordinate structures *via* nearest neighbors in the IB space.

LaTF is further evaluated on a multi-body system, the Lennard-Jones 7 (LJ7) cluster, where seven particles interact *via* the Lennard-Jones potential (see Methods for simulation details).^75,76^ Even though this system has fewer atoms than Chignolin, the FES of LJ7 exhibits richer temperature dependence due to competing energetic and entropic contributions. Using physical order parameters (OPs), such as the second and third moments of coordination numbers (details in SI), we observe clear thermally driven transitions and metastable-state flipping (Fig. 5(a)), highlighting strong entropic effect at high temperature and making LJ7 a valuable test case for LaTF's generation capacity. Trained on data from 0.2*ε*/*k*_B_ and 0.5*ε*/*k*_B_, LaTF identifies four metastable states at low temperature and captures their temperature-dependent shifts in the IB space, consistent with the physical OPs (Fig. 5(c)). Generation KL divergence demonstrates that tilted Gaussians with an appropriate *τ* parameter significantly outperform standard Gaussians in predicting FES across temperatures from 0.2*ε*/*k*_B_ to 0.7*ε*/*k*_B_ (Fig. 5(b)). This advantage arises from their ability to allow both overlap and separation between priors at different temperatures, while standard Gaussians tend to let high-temperature priors fully cover those at lower temperatures, thereby obscuring entropic distinctions. By selecting *τ* based solely on generation error at training temperatures, LaTF generalizes well to unseen temperatures with comparable accuracy, largely enhancing data efficiency. Notably, LaTF captures the temperature-dependent flipping and shifting of FES basins (Fig. 5(d)), though the most populated metastable state corresponding to hexagonal structures is gradually diminished as temperature rises, whereas MD simulations show it should be sharply suppressed. Adopting a more fine-grained *τ* slicing procedure for screening and selection may help address this limitation.

Furthermore, structural ensembles reconstructed from generated IB samples *via* nearest-neighbor matching in the training data yield coordination number moments consistent with MD simulations (Fig. 5(e)). Similar results are obtained when training LaTF with data at 0.2*ε*/*k*_B_ and 0.7*ε*/*k*_B_ (Fig. S8). All above results validate that LaTF exhibits strong transferability, precisely inferring equilibrium distributions at unseen temperatures and significantly improving data efficiency.

### 2.4 Exploring temperature-dependent RNA free energy landscapes with LaTF

We now test LaTF for a significantly more challenging task, *i.e.*, simultaneously identifying dynamically meaningful low-dimensional representations and predicting the temperature response for an RNA system. Unlike proteins, which typically fold into a dominant native state within a funnel-shaped energy landscape,^79,80^ RNAs adopt a highly heterogeneous ensemble of structures with multiple metastable states sharing comparable stability, leading to an intrinsically rugged and disordered energy landscape.^81–84^ This structural complexity, coupled with the significant influence of environmental factors, especially the temperature,^81^ makes RNA an ideal but difficult test case for generative AI methods in molecular sciences. Over the past decade, MD simulations and enhanced sampling methods have been increasingly employed to gain insights into RNA's conformational landscape, though obtaining converged global equilibrium distributions across temperatures remains computationally intensive.^85–88^ Here, we show that LaTF may serve as a potential tool for predicting RNA's temperature dependence in a data-efficient manner.

**Fig. 6:**
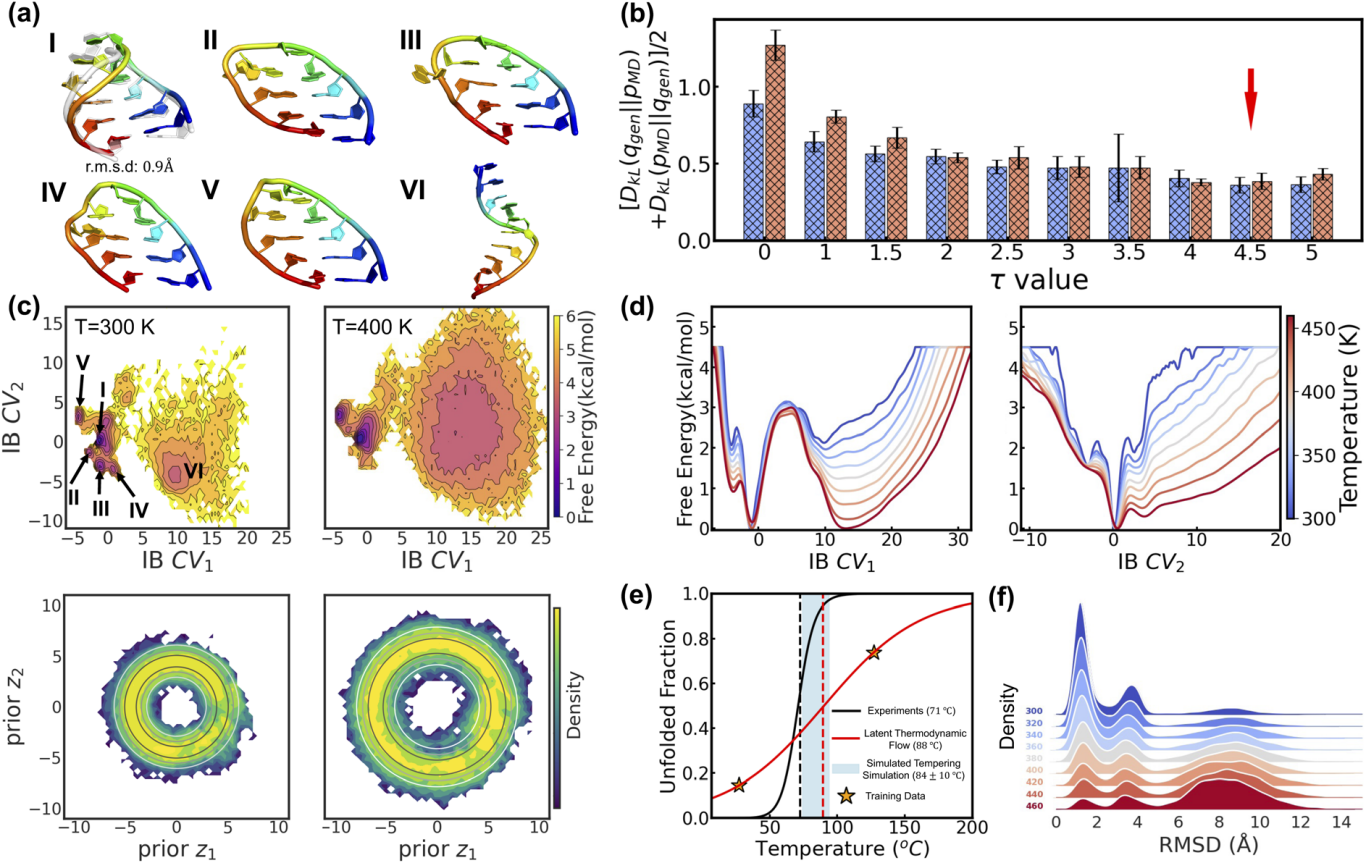
Inferring the temperature dependence of GCAA RNA tetraloop system from data at 300 K and 400 K *via* LaTF. (a) Structure with the highest likelihood scored by LaTF from six metastable states. The NMR structure (gray, transparent) is overlaid with the identified folded structure for comparison. The structures are colored from blue to red from the 5′ to 3′ end. (b) Symmetric KL divergence between LaTF-generated IB distributions and those encoded from validation data. The optimal *τ* = 4.5 (indicated by the red arrow) minimizes the KL divergence. (c) FES in the IB space and associate density in the prior space, estimated from encoded and flow-transformed simulation data. Contours represent the analytical tilted prior, and the density is normalized by the histogram approach. (d) Temperature-dependent FES inferred by LaTF in the IB space across a range of 300 K to 460 K at 20 K intervals. (e) Unfolded fraction over temperatures predicted by LaTF (red), compared with the experimental observations (black)^77^ and simulated tempering results (light blue).^78^ Structural ensembles generated by LaTF are reconstructed using nearest neighbors in IB space from simulation data. A conformation is classified as folded if all three base pairs are formed and its all-atom RMSD to any NMR structure is below 4.0 Å; otherwise, it is considered unfolded. (f) RMSD distributions of LaTF-generated structures relative to the NMR structure across different temperatures.

Specifically, we focus on the ggcGCAAgcc tetraloop (PDB: 1ZIH^89^), a well-characterized system suitable for validation against both experimental and computational references.^77,78,85,87^ Tetraloops are among the most common and functionally important RNA motifs. Despite their small size, they are highly stable and structurally diverse. In this work we model the temperature-dependent structural ensembles for the GCAA tetraloop (Fig. 6(a)) directly from sequence. Secondary structures are first predicted, followed by stepwise modeling of tertiary structures using bioinformatic approaches. The resulting RNA tertiary structures are solvated in explicit water, and simulations are independently performed at 300 K and 400 K. Iterative reseeding of simulations with generated samples from LaTF trained on-the-fly yields total accumulated simulation times of ∼189 µs and ∼148 µs at 300 K and 400 K, respectively. To ensure that the training data used for LaTF is effectively unbiased, we independently construct MSMs at both temperatures from the raw simulation data and use these models to generate long and equilibrium trajectories for training (see Methods for more details).

The equilibrium data are used to train the LaTF model, with tilting factor *τ* = 4.5 selected to perfectly accommodate the data from two temperatures (Fig. 6(b) and (c)). Despite employing a large lag time for training (Δ*t* = 200 ns), we still identify multiple metastable states (six in total) at 300 K (Fig. 6(c)), indicating a multi-modal and hence glassy free energy landscape that persists at long timescales. Representatives for each state are obtained by projecting all MD conformers into the IB space, evaluating their likelihood using the NF, and selecting the most probable structure for each state (Fig. 6(a)). The structure from the most populated state aligns well with the NMR structure from the PDB, showing an all-atom RMSD of 0.9 Å. The four-nucleotide loop is observed to exhibit greater flexibility than the helical region, where even conformational fluctuations of single nucleotides give rise to new metastable states. At 300 K, the unfolded state tends to adopt conformation stabilized by base stacking, whereas at 400 K, increased entropy effect drives the unfolded state into a broader, deeper, and more diverse free energy basin (Fig. 6(c)).

The thermodynamic interpolation ability of LaTF allows inference of temperature-dependent FES in the IB space. As shown in Fig. 6(d), increasing temperature gradually broadens the unfolded regions and destabilizes the folded regions. Since the LaTF-identified CVs effectively separate conformations by their kinetic differences, we use the IB-space-distance as a metric to backmap generated samples to structures of their nearest neighbors in the simulation data. This allows classification of generated conformers into folded or unfolded states and facilitates the derivation of a continuous melting curve for the GCAA tetraloop (Fig. 6(e)). The predicted melting profile aligns well with results from extensive simulated tempering simulations performed using the same force field.^78^ Although both LaTF and prior computational estimates slightly overpredict the experimental melting temperature,^77^ the discrepancy likely arises from factors such as force-field limitations, state definitions, or ionic conditions, *etc.* Meanwhile, the RMSD of generated-and-reconstructed structural ensembles relative to the reference PDB structure across temperatures further supports LaTF's ability to accurately capture the temperature dependence of RNA structural ensembles (Fig. 6(f)).

Notably, the efficient inference of RNA's complex temperature-dependent behaviors emerges from combined strengths of representation learning and generative modeling. Due to RNA's intrinsically rugged energy landscape and the essential role of explicit solvent models in capturing its conformational changes,^90,91^ prior NF-based generative approaches using implicit solvents fall short in accurately modeling such high-dimensional equilibrium distributions, particularly across temperatures.^36,38^ In contrast, LaTF learns compact yet informative representations of the disordered landscape, optimally leveraging explicit-solvent simulation data to precisely model temperature-dependent FES while enabling interpretation and backmapping to all-atom structures.

## 3 Conclusion & discussion

Accurately characterizing the equilibrium distributions of complex molecular systems, as well as their response to environmental factors such as temperature, is essential for uncovering the underlying thermodynamic properties and molecular mechanisms, and projecting these distributions onto physically meaningful low-dimensional representations further facilitates interpretation and understanding. Recent advances in generative AI methods, such as NF models, have shown enormous promise in learning these distributions and providing generative access to configurations. However, in the absence of pre-defined or learnable low-dimensional representations, such methods do not scale well with system size and complexity, limiting their broader applicability. In this work, we present LaTF, a unified end-to-end framework that integrates SPIB with a NF to enable low-dimensional representation learning, metastable state classification, FES estimation, transition pathway interpolation, and inference of temperature-dependent behaviors within a single workflow. We show that both the joint learning of representations and generative models, and the utilization of an expressive tilted Gaussian prior, are critical to LaTF's performance. The former enables the model to better identify kinetically meaningful CVs and enhances both the efficiency and fidelity of generations, while the latter creates a structured and interpretable prior space that supports physically realistic interpolations. The incorporation of a temperature-steerable parameter into the tilted prior further broadens LaTF's applicability, allowing it to predict nontrivial thermodynamic responses from limited data. Applications to diverse systems, including a model potential, protein, multi-body particles, and RNA, demonstrate LaTF's accuracy and utility.

It is worth noting that there is still room for improvement in the LaTF framework. For instance, all our tested cases empirically suggest that training LaTF with data collected at different temperatures works well. However, there is no theoretical guarantee that the learned representations themselves should remain unchanged with temperature. Although SPIB has demonstrated robustness and can capture essential degrees of freedom even when trained on biased data from enhanced sampling,^25^ additional steps such as reweighting data or rescaling time steps across temperatures may further enhance its performance.^27,92^ In our implementation, temperature dependence is introduced through linear interpolation of a steerable parameter in the tilted Gaussian prior, which yields numerically reasonable predictions of temperature-dependent FES across different systems. Still, allowing this parameter to be learnable or to serve as an input to the network may provide greater flexibility and further enhance interpolation and extrapolation performance.^38,43^ Meanwhile, making tilting parameter *τ* a low-complexity, temperature-dependent parameter may also be beneficial. This can be implemented using a simple linear neural network layer or, as demonstrated in a previous study,^74^ by incorporating a sinusoidal embedding layer for temperature. And setting the prior as a mixture of tilted Gaussian distributions may further enhance the generative model's expressiveness and fidelity.

Additionally, we believe that incorporating more rigorous theoretical grounding or improved computational procedures could further enhance LaTF's ability to infer temperature-dependent distributions. For instance, a more careful quantification of the entropy loss associated with structural descriptor selection and dimensionality reduction^93^ may help guide the design of model architectures and prior structures that more accurately recover temperature-dependent FES. Moreover, while we currently reconstruct structural ensembles by mapping generated latent configurations to their nearest neighbors, an additional reweighting step, *i.e.*, using implicit-solvent force fields to evaluate the potential energy and corresponding equilibrium weights of the generated structures,^36,38^ could improve accuracy, particularly for the LJ7 system, where high-temperature predictions overrepresent the hexagonal metastable state. For systems with non-negligible solvent effects, such as RNA, coupling structure generation with Metropolis-adjusted Langevin dynamics for solvent degrees of freedom, and accepting or rejecting configurations based on the total potential energy,^94,95^ presents a promising future direction.

Beyond the implementations presented in this work, the LaTF framework holds promise for broader applications. For example, it may be extended to study systems exhibiting complex phase behaviors or glassy transitions across a range of environmental conditions, and this would likely require more careful design of the prior distribution.^44,96^ Training LaTF with (reweighted) data from enhanced sampling methods such as replica exchange molecular dynamics trajectories could further improve data efficiency. Moreover, the proposed joint learning framework is not limited to SPIB and NF, alternative dynamical autoencoders such as time-lagged autoencoders,^21^ variational dynamics encoders,^22^ extended autoencoder^97^ and EncoderMap,^98^ as well as generative models like flow matching^99^ or diffusion models,^100,101^ could be incorporated in a similar architecture. We view LaTF as a foundational approach for unified representation learning and generative modeling, upon which a range of future models and applications can be built and extended depending on specific goals and system types.

## 4 Methods

### 4.1 RealNVP normalizing flows

NF adopts neural networks to construct learnable, invertible mappings. The specific implementation, including the network architecture and transformation formula, is highly flexible. We employ a sequence of real-valued non-volume preserving (RealNVP) transformations 
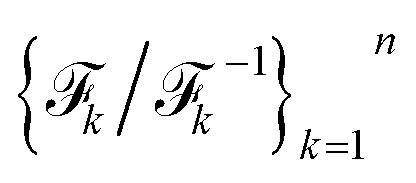
 to build the NF.^55^ RealNVP partitions the encoded IB variables into two channels, (*µ*_1_, *µ*_2_), and applies a series of invertible operations (multiplication and addition) to one channel while keeping the other fixed (Fig. 1). Nonlinearity of the transformation is introduced by using neural networks to parameterize the scaling and translation functions, *S*_*θ*_ and *T*_*θ*_, respectively:6
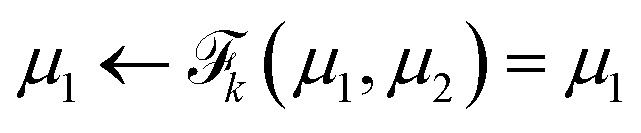
7
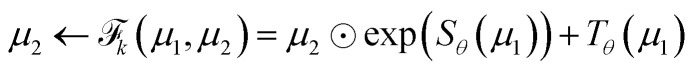


In the subsequent transformation, *µ*_2_ remains fixed while *µ*_1_ is updated. This alternating scheme ensures that the overall Jacobian matrix remains triangular, allowing efficient computation of the determinant. With an analytical prior and tractable Jacobian, the likelihood of any sample in the IB space can be explicitly evaluated. More details of NF and RealNVP are included in the SI.

### 4.2 Exponentially tilted Gaussian distribution

The normalization factors for the tilted Gaussian prior, *Z*_*τ*_, and the temperature-steerable tilted Gaussian prior, *Z*_*τ*,*T*_, can be expressed analytically as:8
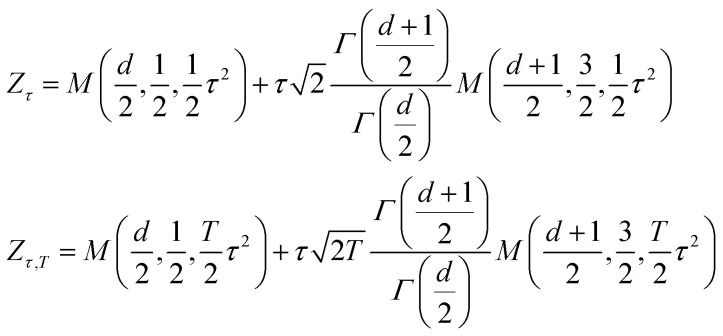


where 
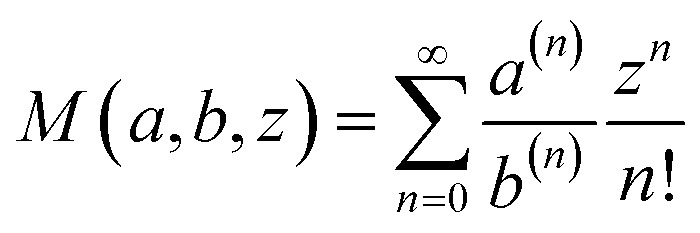
 is the Kummer confluent hypergeometric function, and *a*^(*n*)^ = *a*(*a* + 1) … (*a* + *n* − 1) denotes the rising factorial. Samples from these priors are drawn using Metropolis Monte Carlo sampling. We explicitly evaluate the impact of using an exponentially tilted Gaussian prior on the expressive power of the RealNVP model. Prior work has shown that RealNVP struggles to approximate multi-modal distributions separated by high barriers.^94^ Using the same analytical target distributions, we compare RealNVP performance when equipped with either a standard Gaussian prior or the tilted Gaussian prior. As shown in Fig. S1, the tilted Gaussian prior improves generation accuracy and, to some extent, mitigates mode-collapse. More details on the tilted Gaussian distribution and comparison results are provided in the SI.

### 4.3 Details of molecular dynamics simulations

We adopt the analytical form of the three-hole potential system from ref. 102. Langevin dynamics of a unit-mass particle on this surface is simulated with a time step of 0.001, a temperature setting of 1/*k*_B_, and a friction coefficient of 0.5 per step. The simulation runs for 5 × 10^7^ steps, recording coordinates every 50 steps, resulting in a trajectory of 10^6^ frames (see SI for more details).

For the Chignolin protein, simulations are initialized from the NMR structure (PDB: 1UAO) in explicit solvent, followed by energy minimization and multi-step equilibration, with and without positional restraints. The protein is modeled using the OPLS-AA force field,^103,104^ and the TIP3P water model^105^ is employed. Six independent, long-time unbiased MD simulations are performed for tens of microseconds at six different temperatures, ranging from 340 K to 440 K. The observation of more than twenty reversible folding–unfolding transitions in each trajectory indicates sufficient sampling (simulation details and trajectory lengths are provided in SI).

For the LJ7 system, we simulate seven identical particles in 2D space interacting *via* the Lennard-Jones potential. Six independent simulations are performed at temperatures ranging from 0.2*ε*/*k*_B_ to 0.7*ε*/*k*_B_, using a Langevin thermostat with a friction coefficient of 
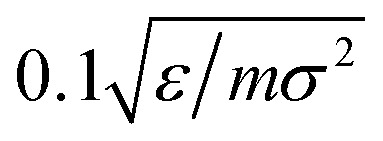
. Each simulation runs for 10^7^ steps, with particle coordinates recorded every 100 steps, yielding trajectories of 10^5^ snapshots per temperature (see SI for more details).

For the RNA ggcGCAAgcc tetraloop system, we begin with the sequence alone and predict corresponding secondary structures using ViennaRNA,^106,107^ followed by tertiary structure modeling using the FARFAR method in Rosetta.^108–110^ In unbiased simulations, RNA is modeled with the DESRES-AMBER force field,^78^ solvated in TIP4P-D water,^111^ and neutralized with 1 M KCl, following the setup from prior work.^78^ Before production runs, the systems undergo energy minimization and stepwise equilibration with and without positional restraints. Hundreds of microseconds simulations are performed at 300 K and 400 K over a few adaptive iterations. After each iteration, an LaTF model is trained using the accumulated data to identify high-likelihood conformers and transition-region structures for reseeding the next round of simulations. Final trajectories are reweighted using validated MSMs to ensure that the data used for LaTF training are both thermodynamically and kinetically unbiased. Full details on structural modeling, simulation procedures, adaptive sampling, and MSMs construction and validation are provided in the SI.

### 4.4 Single-temperature LaTF training

The LaTF training procedure follows that of SPIB,^24,32^ with the key difference being the omission of the on-the-fly VampPrior update, which is replaced by a more expressive NF model (see pseudocode in SI). Training LaTF at a single temperature requires molecular descriptors and an initial set of state labels as input. For the model potential system, the *x*–*y* coordinates are used as input, and initial labels are generated *via* K-means clustering. For the Chignolin protein (340 K), we use pairwise distances between carbon-alpha atoms as descriptors and initialize labels through projection onto Time-lagged Independent Components^61,62^ followed by K-means clustering. During training, LaTF iteratively merges short-lived states to make their lifetimes longer than the lag time Δ*t* and relabels input configurations to improve state metastability. We find that a two-step training strategy, *i.e.*, first training SPIB to obtain a converged state assignment, followed by joint training of SPIB and NF, largely reduces training time and effort. This approach is recommended and used in this study; and we confirm that it yields results consistent with end-to-end joint training for all systems presented in this study.

For both systems, the long simulation trajectory is uniformly divided into five segments, with four used for training and the remaining one reserved for validation. The KL divergence between the generated latent distribution and that encoded from the validation dataset is used to select the optimal tilting factor *τ* for the prior. The KL divergence is computed using a histogram-based approach, with 100 bins evenly spaced between the minimum and maximum values along each IB coordinate. The probability in each bin is estimated and normalized, and a small epsilon value is added to ensure a minimum probability of 1 × 10^−5^, preventing numerical instability. We note that assessing distribution calibration using alternative methods, such as the Fasano–Franceschini test,^112^ can also be beneficial. We apply this test to the 2D analytical potential and Chignolin protein folding systems and find that it yields qualitatively consistent results (see Fig. S13). After training the LaTF models, all simulation data are assigned to metastable states based on the highest decoding probability, allowing construction and evaluation of corresponding MSMs using the GMRQ score.^65^ Additional details on feature extraction, label initialization, neural network architecture, and training hyperparameters are provided in the SI.

### 4.5 Multi-temperature LaTF training

The primary distinction between multi-temperature and single-temperature training lies in the inclusion of an additional input: the temperature-steerable parameter assigned to each configuration. We define this parameter such that the lowest training temperature is set to one, with other temperatures rescaled proportionally. While alternative normalizations may also be viable, the optimal tilting factor *τ* in the tilted Gaussian prior should then be adjusted to minimize the KL divergence between the generated and validation-data-encoded latent distributions.

The Chignolin system is featurized using pairwise distances between carbon-alpha atoms, while the RNA tetraloop is represented by ***r***-vectors^113^ and selected pairwise carbon distances. For both systems, initial labels are generated by applying Principal Component Analysis to multi-temperature data, followed by K-means clustering. In the LJ7 system, each configuration is described by sorted coordination numbers, and initial labels are assigned based on K-means clustering over their second and third moments. For all cases, the dataset is uniformly split into five partitions, with four used for training and one for validation. More details on feature construction, label initialization, network architecture, and training hyperparameters are provided in the SI.

## Author contributions

P. T. and Y. Q. conceptualized the study, with P. T. providing supervision. Y. Q. developed the methodology and, together with R. J., designed the computational experiments. Y. Q. conducted the simulations, and Y. Q. and R. J. performed the data analysis. Y. Q., R. J., and L. H. interpreted the results. Y. Q. and R. J. prepared the initial draft of the manuscript, and all authors contributed to reviewing, editing, and approving the final version.

## Conflicts of interest

The authors declare no competing interest.

## Supplementary Material

SC-017-D5SC06402C-s001

## Data Availability

The simulation datasets for the Chignolin protein and RNA tetraloop generated in this work can be obtained from authors upon request. The full source code of the Latent Thermodynamic Flows model, along with simulation data for the 2D analytical potential and Lennard-Jones particle systems and accompanying documentation, is publicly accessible at: https://github.com/tiwarylab/LatentThermoFlows. Supplementary information: Supporting Information (SI) providing detailed descriptions of the unified loss function derivation, the tilted Gaussian prior, the normalizing flow models, and the simulation and model training procedures, is available. See DOI: https://doi.org/10.1039/d5sc06402c.
