# Supplementary material for: Latent thermodynamic flows: unified representation learning and generative modeling of temperature-dependent behaviors from limited data
Source: Chem Sci. 2026 Jan 2;17(7):3743–57. doi: 10.1039/d5sc06402c (PMC12758490; doi:10.1039/d5sc06402c)
Supplement: SC-017-D5SC06402C-s001 [file SC-017-D5SC06402C-s001.pdf]

Supplementary Information (SI) for Chemical Science.
This journal is © The Royal Society of Chemistry 2025

Supplementary Information: Latent Thermodynamic Flows: Unified Representation Learning and Generative Modeling of Temperature-Dependent Behaviors from Limited Data

Yunrui Qiu,[1,2] Richard John,[1,3] Lukas Herron,[1,4,2] and Pratyush Tiwary *[1,2,5]

[1]Institute for Physical Science and Technology, University of Maryland, College Park, MD, 20742, USA
[2]Institute for Health Computing, University of Maryland, Bethesda, MD, 20852, USA
[3]Department of Physics, University of Maryland, College Park, MD, 20742, USA
[4]Biophysics Program, University of Maryland, College Park, MD, 20742, USA
[5]Department of Chemistry and Biochemistry, College Park, MD, 20742, USA

Contents

I. A Unified Loss Framework for Latent Thermodynamic Flows 2
A. Information Bottleneck (IB) 2
B. State Predictive Information Bottleneck (SPIB) 3
C. Latent Thermodynamic Flows (LaTF) 3

II. Exponentially tilted Gaussian Distribution 5

III. Normalizing Flows and Real NVP Transformations 7

IV. Details of Molecular Dynamics Simulations 9
A. Three-hole Potential System 9
B. Chignolin Protein 9
C. Lennard-Jones 7 System 10
D. GCAA RNA Tetraloops 10

V. Training of Latent Thermodynamic Flows 12
A. Feature Extraction and Initialization of State Labels 12
B. Training Procedure 13
C. Network Architecture and Training Hyperparameters 13
D. Choice of Information Bottleneck Dimensionality 15
E. Interpolation of Transition Pathways 17

References 32

* Corresponding author:ptiwary@umd.edu

I. A UNIFIED LOSS FRAMEWORK FOR LATENT THERMODYNAMIC FLOWS

The State Predictive Information Bottleneck (SPIB)[1] has been developed and validated as a state-of-the-art method for the systematic analysis of molecular dynamics (MD) data across a variety of systems[2–4]. Grounded in the Information Bottleneck (IB) principle[5, 6], SPIB simultaneously learns an informative low-dimensional latent representation and identifies long-lived metastable states. Here, we integrate a generative model, specifically a normalizing flow, into the latent space, which can be trained jointly with SPIB. The low-dimensional representation allows the generative model to focus its expressivity on the most meaningful attributes of the data and accelerates sample generation. On the other hand, the generative model enables accurate quantification of the latent distribution, thereby improving the optimization of the latent space. Furthermore, we find that, with an informative prior, the latent generative model exhibits strong generative capability in reproducing free energy landscapes across a broad range of temperatures despite being trained on limited data from only a few temperature conditions. We refer to the whole framework, including both representation learning and generative modeling, as Latent Thermodynamic Flows (LaTF). This section provides a brief overview of the IB principle, reviews the objective function for SPIB, and presents a detailed derivation for the loss function of LaTF.

A. Information Bottleneck (IB)

The IB principle is designed to extract a low-dimensional representation $\boldsymbol{z}$ from high-dimensional input data $\boldsymbol{X} \in \{\boldsymbol{X}^n\}_{n=1}^N$ that retains the most relevant information to predict a target variable $\boldsymbol{y} \in \{\boldsymbol{y}^n\}_{n=1}^N$. More formally, we presuppose some conditional probability distribution $p_\theta(\boldsymbol{z}|\boldsymbol{X})$, parameterized by θ, which we call an *encoder*. The IB principle consists of maximizing the following objective with respect to the encoder parameters:

$$\underset{\theta}{argmax}\ \mathcal{L}_{IB} = I(\boldsymbol{z}, \boldsymbol{y}; \theta) - \beta I(\boldsymbol{X}, \boldsymbol{z}; \theta) \tag{1}$$

where $I(x, y) = \int dxdy\ p(x, y) \log \frac{p(x,y)}{p(x)p(y)}$ denotes mutual information, which quantifies the shared information between the variables x and y. Models maximizing this objective function thus seek to determine a latent representation $\boldsymbol{z}$ that balances being maximally informative when predicting $\boldsymbol{y}$, but relies on the minimum amount of information about $\boldsymbol{X}$, a tradeoff mediated by a hyperparameter $\beta \in [0, +\infty)$.

Due to the high computational cost of directly quantifying mutual information, a variational lower bound is commonly employed as an alternative objective function. To motivate this variational lower bound, we first note that for any probability distributions $q(\boldsymbol{y}|\boldsymbol{z})$ and $r(\boldsymbol{z})$, the following inequality must be satisfied due to the positivity of the Kullback-Leibler divergence and Markovianity conditions on $\boldsymbol{X}$, $\boldsymbol{y}$, and $\boldsymbol{z}$:

$$\mathcal{L}_{IB} \geq \mathcal{L} = \int d\boldsymbol{X} d\boldsymbol{y} d\boldsymbol{z} \Big[p(\boldsymbol{X}) p(\boldsymbol{y}|\boldsymbol{X}) p_\theta(\boldsymbol{z}|\boldsymbol{X}) \log q(\boldsymbol{y}|\boldsymbol{z})\Big] - \beta \int d\boldsymbol{X} d\boldsymbol{z} \Big[p(\boldsymbol{X}) p_\theta(\boldsymbol{z}|\boldsymbol{X}) \log \frac{p(\boldsymbol{z}|\boldsymbol{X})}{r(\boldsymbol{z})}\Big] + \mathcal{H}(\boldsymbol{y}) \tag{2}$$

where the entropy $\mathcal{H}(\boldsymbol{y}) = -\int dy\ p(\boldsymbol{y}) \log p(\boldsymbol{y})$ is a constant and can be omitted during the optimization process. We may now parameterize the *decoder* $q_\theta(\boldsymbol{y}|\boldsymbol{z})$ and *prior* $r(\boldsymbol{z})$ distributions in preparation for maximizing this lower bound on the true objective. Finally, we may eliminate the inaccessible distributions $p(\boldsymbol{X})$ and $p(\boldsymbol{y}|\boldsymbol{X})$ by approximation via the empirical distribution $p(\boldsymbol{X}, \boldsymbol{y}) = \frac{1}{N} \sum_n \delta_{\mathbf{X}^n}(\boldsymbol{X}) \delta_{\boldsymbol{y}^n}(\boldsymbol{y})$. The final variational lower bound to the information bottleneck objective function is as follows:

$$\mathcal{L}_{IB} \geq \mathcal{L} \approx -\frac{1}{N} \sum_{n=1}^{N} \int d\boldsymbol{z} \Big[-p_\theta(\boldsymbol{z}|\boldsymbol{X}^n) \log(q_\theta(\boldsymbol{y}^n|\boldsymbol{z}))\Big] - \beta \frac{1}{N} \sum_{n=1}^{N} \int d\boldsymbol{z} \Big[p_\theta(\boldsymbol{z}|\boldsymbol{X}^n) \log \frac{p_\theta(\boldsymbol{z}|\boldsymbol{X}^n)}{r(\boldsymbol{z})}\Big] \tag{3}$$

which clearly depends only on the distributions $p_\theta(\boldsymbol{z}|\boldsymbol{X})$, $q_\theta(\boldsymbol{y}|\boldsymbol{z})$ and $r(\boldsymbol{z})$, and empirical data $\{\boldsymbol{X}^n, \boldsymbol{y}^n\}$. Thus, by appropriately choosing analytical forms for these three distributions and optimizing the entire objective with respect to θ, we arrive at a latent variable $\boldsymbol{z}$ satisfying the information bottleneck principle.

B. State Predictive Information Bottleneck (SPIB)

The time-sequential nature of MD data inspired the extension of the IB framework into SPIB. In line with the IB principle, SPIB aims to extract the most predictive features from molecular structures, enabling accurate inference of their future evolution. Specifically, the inputs to SPIB are high-dimensional structural descriptors representing MD conformations, and the outputs are labels corresponding to the metastable state into which the conformation is expected to transit after a lag time Δt. As a result, the low-dimensional bottleneck space typically integrates structural descriptors with strong time-lagged correlations and effectively captures the essential slowest dynamical motions. In particular, SPIB employs deep neural networks to parameterize the probability distributions $p_\theta(\boldsymbol{z}|\boldsymbol{X})$, $q_\theta(\boldsymbol{y}|\boldsymbol{z})$ and $r_\theta(\boldsymbol{z})$, and is typically trained on unbiased MD trajectories, with state labels derived from clustering strategies. For instance, given an unbiased trajectory $\{\boldsymbol{X}^{dt}, \boldsymbol{X}^{2dt}, \cdots, \boldsymbol{X}^{Mdt}, \cdots, \boldsymbol{X}^{(M+s)dt}\}$ and the corresponding state labels $\{\boldsymbol{y}^{dt}, \boldsymbol{y}^{2dt}, \cdots, \boldsymbol{y}^{Mdt}, \cdots, \boldsymbol{y}^{(M+s)dt}\}$, where $s \cdot dt = \Delta t$, the objective function of SPIB is expressed as:

$$\underset{\theta}{argmax}\ \mathcal{L} = \frac{1}{M}\sum_{n=1}^{M}\int d\boldsymbol{z}^n p_\theta(\boldsymbol{z}^n|\boldsymbol{X}^n)\Big[\log q_\theta(\boldsymbol{y}^{n+s}|\boldsymbol{z}^n) - \beta \log \frac{p_\theta(\boldsymbol{z}^n|\boldsymbol{X}^n)}{r(\boldsymbol{z}^n)}\Big]. \tag{4}$$

The first term, quantifying the predictive accuracy of the target state, is evaluated using a decoder neural network $\mathbb{D}$ with softmax outputs, where the conditional probability $q_\theta(\boldsymbol{y}^{n+s}|\boldsymbol{z}^n)$ is computed as $\sum_{i=1}^{k} \boldsymbol{y}_i^{n+s} \log \mathbb{D}_i(\boldsymbol{z}^n)$, with $\boldsymbol{y}$ denoting an k-dimensional one-hot state label vector. The second term acts as a regularization term, measuring the discrepancy between the prior distribution and the encoded posterior. Specifically, a Gaussian encoder $\mathbb{E}$ and a modified variational mixture of posteriors prior are employed, i.e., $p_\theta(\boldsymbol{z}^n|\boldsymbol{X}^n) = \mathcal{N}(\boldsymbol{z}^n; \mathbb{E}_1(\boldsymbol{X}^n), \mathbb{E}_2(\boldsymbol{X}^n)\boldsymbol{I})$, $r(\boldsymbol{z}) = \sum_{i=1}^{k} \omega_i p_\theta(\boldsymbol{z}|\boldsymbol{X}_{rep}^i)$. The encoded posterior distribution is modeled as a Gaussian, parameterized by the transformed input representation, while the prior distribution is a weighted linear combination of posteriors from representative inputs, subject to the constraint that the weights sum to one ($\sum_{i=1}^{k} \omega_i = 1$). In practice, SPIB is trained using an iterative, self-consistent scheme, wherein the number and positions of states are updated dynamically to enhance state metastability. This setup ensures that each configuration is likely to remain within the same state over the specified lag time Δt.

C. Latent Thermodynamic Flows (LaTF)

Within the SPIB framework, the latent space distribution is regularized using a mixture of Gaussian components, which has been shown to improve model expressivity and promote a more structured latent representation compared to the conventional isotropic Gaussian prior. However, the assumptions of a Gaussian encoder and a mixed Gaussian prior are strong and often difficult to justify for complex data such as MD trajectories, thereby hindering accurate characterization of the latent distribution. Here, inspired by prior studies[7–10], we integrate a flow-based model into the SPIB framework to establish an invertible transformation between the latent posterior distribution and a tractable, simple prior distribution, thereby enabling more accurate quantification of the latent space distribution. This unified approach enables simultaneous learning of both informative representations and their associated distributions, further facilitating inference across varying thermodynamic conditions.

Specifically, we employ a normalizing flow $\mathcal{F}_\theta$ to construct the posterior distribution by transforming a random variable $\boldsymbol{u}$, which depends on encoded $\boldsymbol{X}$, into the target latent variable $\boldsymbol{z}$, thereby avoiding the need for an explicitly defined analytical form. The model architecture is illustrated in Figure 1 of the main text. Mathematically, the invertible mapping defined by the normalizing flow can be expressed using an integral involving the Dirac delta function:

$$p_\theta(\boldsymbol{z}|\boldsymbol{X}^n) = \int d\boldsymbol{u}\ \delta\Big(\boldsymbol{z} - \mathcal{F}_\theta(\boldsymbol{u})\Big) p_\theta(\boldsymbol{u}|\boldsymbol{X}^n) \tag{5}$$

Substituting the revised expression for the posterior distribution into the SPIB loss function yields:

$$\mathcal{L}_{LaTF} = \frac{1}{M}\sum_{n=1}^{M}\int d\boldsymbol{z}^n \left[p_\theta(\boldsymbol{z}^n|\boldsymbol{X}^n)\log\frac{q_\theta(\boldsymbol{y}^{n+s}|\boldsymbol{z}^n)\cdot r^\beta(\boldsymbol{z}^n)}{p_\theta^\beta(\boldsymbol{z}^n|\boldsymbol{X}^n)}\right] \tag{6}$$

$$= \frac{1}{M}\sum_{n=1}^{M}\iint d\boldsymbol{z}^n d\boldsymbol{u}\left[\delta(\boldsymbol{z}^n - \mathcal{F}_\theta(\boldsymbol{u}))p_\theta(\boldsymbol{u}|\boldsymbol{X}^n)\log\frac{q_\theta(\boldsymbol{y}^{n+s}|\boldsymbol{z}^n)\cdot r^\beta(\boldsymbol{z}^n)}{(\int d\boldsymbol{u}'\delta(\boldsymbol{z}^n - \mathcal{F}_\theta(\boldsymbol{u}'))p_\theta(\boldsymbol{u}'|\boldsymbol{X}^n))^\beta}\right] \tag{7}$$

$$= \frac{1}{M}\sum_{n=1}^{M}\int d\boldsymbol{u}\; p_\theta(\boldsymbol{u}|\boldsymbol{X}^n)\log\frac{q_\theta(\boldsymbol{y}^{n+s}|\mathcal{F}_\theta(\boldsymbol{u}))\cdot r^\beta(\mathcal{F}_\theta(\boldsymbol{u}))}{(\int d\boldsymbol{u}'\delta(\mathcal{F}_\theta(\boldsymbol{u}) - \mathcal{F}_\theta(\boldsymbol{u}'))p_\theta(\boldsymbol{u}'|\boldsymbol{X}^n))^\beta} \tag{8}$$

Let $\boldsymbol{h}' = \mathcal{F}_\theta(\boldsymbol{u}')$ and $\boldsymbol{u}'(\boldsymbol{X}^n) = \mathcal{F}_\theta^{-1}(\boldsymbol{h}')$, so that the determinant of the Jacobian matrix associated with this invertible transformation can be expressed as: $\det[\frac{\partial \boldsymbol{h}'}{\partial \boldsymbol{u}'}] = \det[\frac{\partial \mathcal{F}_\theta(\boldsymbol{u}'|\boldsymbol{X}^n)}{\partial \boldsymbol{u}'}]$. One may further simplify the loss function:

$$\mathcal{L}_{LaTF} = \frac{1}{M}\sum_{n=1}^{M}\int d\boldsymbol{u}\; p_\theta(\boldsymbol{u}|\boldsymbol{X}^n)\log\frac{q_\theta(\boldsymbol{y}^{n+s}|\mathcal{F}_\theta(\boldsymbol{u}))\cdot r^\beta(\mathcal{F}_\theta(\boldsymbol{u}))}{(\int d\boldsymbol{h}'\;|\det[\frac{\partial \boldsymbol{u}'}{\partial \boldsymbol{h}'}]|\;\delta(\mathcal{F}_\theta(\boldsymbol{u}) - \boldsymbol{h}')\;p_\theta(\mathcal{F}_\theta^{-1}(\boldsymbol{h}'))^\beta} \tag{9}$$

$$= \frac{1}{M}\sum_{n=1}^{M}\int d\boldsymbol{u}\; p_\theta(\boldsymbol{u}|\boldsymbol{X}^n)\log\frac{q_\theta(\boldsymbol{y}^{n+s}|\mathcal{F}_\theta(\boldsymbol{u}))\cdot r^\beta(\mathcal{F}_\theta(\boldsymbol{u}))}{(|\det[\frac{\partial \boldsymbol{u}}{\partial \boldsymbol{h}}]|\;p_\theta(\mathcal{F}_\theta^{-1}(\mathcal{F}_\theta(\boldsymbol{u})))^\beta} \tag{10}$$

$$= \frac{1}{M}\sum_{n=1}^{M}\int d\boldsymbol{u}\; p_\theta(\boldsymbol{u}|\boldsymbol{X}^n)\log\frac{q_\theta(\boldsymbol{y}^{n+s}|\mathcal{F}_\theta(\boldsymbol{u}))\cdot r^\beta(\mathcal{F}_\theta(\boldsymbol{u}))(|\det[\frac{\partial \mathcal{F}_\theta(\boldsymbol{u})}{\partial \boldsymbol{u}}]|)^\beta}{(p_\theta(\boldsymbol{u}|\boldsymbol{X}^n))^\beta} \tag{11}$$

$$= \frac{1}{M}\sum_{n=1}^{M}\int d\boldsymbol{u}\; p_\theta(\boldsymbol{u}|\boldsymbol{X}^n)\log\left[q_\theta(\boldsymbol{y}^{n+s}|\mathcal{F}_\theta(\boldsymbol{u}))\cdot r^\beta(\mathcal{F}_\theta(\boldsymbol{u}))\cdot(|\det[\frac{\partial \mathcal{F}_\theta(\boldsymbol{u})}{\partial \boldsymbol{u}}]|)^\beta\right] + const. \tag{12}$$

$$= \frac{1}{M}\sum_{n=1}^{M}\int d\boldsymbol{u}\; p_\theta(\boldsymbol{u}|\boldsymbol{X}^n)\left[\log q_\theta(\boldsymbol{y}^{n+s}|\mathcal{F}_\theta(\boldsymbol{u})) + \beta\log r(\mathcal{F}_\theta(\boldsymbol{u})) + \beta\log|\det[\frac{\partial \mathcal{F}_\theta(\boldsymbol{u})}{\partial \boldsymbol{u}}]|\right] + const. \tag{13}$$

Following the setup used in SPIB, we define the latent random variable $\boldsymbol{u}$ using a Gaussian encoder $\mathbb{E}$, such that $p_\theta(\boldsymbol{u}|\boldsymbol{X}^n) = \mathcal{N}(\boldsymbol{u}; \mathbb{E}(\boldsymbol{X}^n), \sigma_\theta \boldsymbol{I})$, and apply the normalizing flow transformation as $\mathcal{F}_\theta(\boldsymbol{u}) = \mathcal{F}_\theta(\mathbb{E}(\boldsymbol{X}^n) + \sigma_\theta \cdot \boldsymbol{\xi})$, where $\boldsymbol{\xi}$ is standard Gaussian noise. The normalizing flow is used to refine the encoded distribution to better match the target prior. Furthermore, the distribution $q_\theta(y^{n+s}|\mathcal{F}_\theta(\boldsymbol{u}))$ could be similarly evaluated using a reversible flow and Gaussian decoder, expressed as $\sum_{i=1}^{k}\boldsymbol{y}_i^{n+s}\log\mathbb{D}_i(\mathcal{F}_\theta^{-1}(\mathcal{F}_\theta(\boldsymbol{u})))$, and the prior $r(\mathcal{F}_\theta(\boldsymbol{u}))$ is modeled with a more general form known as the exponentially tilted Gaussian (see details in Sec. II).

Considering a special case, if the invertible transformation is chosen to be a simple linear mapping, i.e.,$\mathcal{F}(\boldsymbol{u}|\boldsymbol{X}^n) = \boldsymbol{\mu}(\boldsymbol{X}^n) + \boldsymbol{\sigma}(\boldsymbol{X}^n)\otimes\boldsymbol{u}$, this linear form corresponds to the reparameterization trick, effectively reducing the model to a standard variational autoencoder (VAE). In this case, the determinant of the Jacobian matrix simplifies to:

$$\mathbb{E}_{\boldsymbol{u}\sim p(\boldsymbol{u})}[|\det[\frac{\partial \mathcal{F}(\boldsymbol{u}|\boldsymbol{X}^n)}{\partial \boldsymbol{u}}]|] = -\sum_{i=1}^{dim}\log\sigma_i(\boldsymbol{X}^n) \tag{14}$$

thereby aligning the resulting loss function with that of the canonical VAE[6].

II. EXPONENTIALLY TILTED GAUSSIAN DISTRIBUTION

To enhance the model's ability to accurately approximate the latent space distribution and make the model more expressive, we adopt a generalized analytical prior known as the exponentially tilted Gaussian[11]. The conventional isotropic Gaussian probability density function p_0 in a $\mathbb{R}^{d_z}$ space is given by:

$$r_0(\boldsymbol{z}) = \frac{1}{(2\pi)^{\frac{d_z}{2}}\det(\Sigma)^{\frac{1}{2}}} \cdot \exp(-\frac{1}{2}(\boldsymbol{z}-\boldsymbol{\mu})^T\Sigma^{-1}(\boldsymbol{z}-\boldsymbol{\mu})) \tag{15}$$

where $\boldsymbol{\mu}$ and Σ denote the mean vector and covariance matrix, respectively. By applying an exponential tilting to the isotropic Gaussian distribution based on its norm, with tilting parameter τ, the resulting probability density function $r(\boldsymbol{z},\tau)$ in $\mathbb{R}^{d_z}$ space is defined as:

$$r(\boldsymbol{z},\tau) = \frac{\exp(\tau||\boldsymbol{z}||)}{Z_\tau} \cdot \frac{\exp(-\frac{1}{2}||\boldsymbol{z}||^2)}{(2\pi)^{\frac{d_z}{2}}} = \frac{\exp(\tau||\boldsymbol{z}||)}{Z_\tau} \cdot r_0(\boldsymbol{z}) \tag{16}$$

$$= \frac{1}{Z_\tau(2\pi)^{\frac{d_z}{2}}} \cdot \exp(-\frac{1}{2}(||\boldsymbol{z}||-\tau)^2) \cdot \exp(\frac{1}{2}\tau^2) \tag{17}$$

where $Z_\tau = \mathbb{E}_{\boldsymbol{z}\sim\mathcal{N}(\boldsymbol{0},\boldsymbol{I})}[\exp(\tau||\boldsymbol{z}||)]$ is the normalization constant of the distribution. The standard Gaussian distribution emerges as a special case of the tilted Gaussian when the tilting parameter is set to $\tau = 0$. Notably, by completing the square as shown in Equation 17, the tilted Gaussian is revealed to be radially symmetric, attaining its maximum at $|\boldsymbol{z}| = \tau$. The normalization constant for the distribution can be analytically calculated as[11]:

$$Z_\tau = \mathbb{E}_{\boldsymbol{z}\sim\mathcal{N}(\boldsymbol{0},\boldsymbol{I})}[e^{\tau||\boldsymbol{z}||}] = \int_{\mathbb{R}^{d_z}} e^{\tau||\boldsymbol{z}||} \cdot \frac{\exp(-\frac{1}{2}||\boldsymbol{z}||^2)}{(2\pi)^{-\frac{d_z}{2}}}\, d\boldsymbol{z} \tag{18}$$

$$= \sum_{n\ even}^{\infty} \frac{\tau^n d(d+2)\cdots(d+n-2)}{n!} + \sum_{n\ odd}^{\infty} \frac{\tau^n \mu_1(d+1)(d+3)\cdots(d+n-2)}{n!} \quad (\mu_1 = \sqrt{2}\frac{\Gamma(\frac{d+1}{2})}{\Gamma(\frac{d}{2})}) \tag{19}$$

$$= M(\frac{d}{2},\frac{1}{2},\frac{1}{2}\tau^2) + \tau\sqrt{2}\frac{\Gamma(\frac{d+1}{2})}{\Gamma(\frac{d}{2})}M(\frac{d+1}{2},\frac{3}{2},\frac{1}{2}\tau^2) \tag{20}$$

where $M(a,b,z) = \sum_{n=0}^{\infty}\frac{a^{(n)}}{b^{(n)}}\frac{z^n}{n!}$ denotes the Kummer confluent hypergeometric function, while $a^{(n)} = a(a+1)\cdots(a+n-1)$ represents the rising factorial.

Employing a more structured tilted Gaussian as the prior distribution for the Normalizing Flows (NF) model increases the volume of high-probability regions, enabling the model to more effectively accommodate and distinguish data originating from multiple modes. We explicitly compare this prior with the standard normal Gaussian prior within a Real-valued Non-Volume Preserving (RealNVP) NF architecture (see more details in the next section) to demonstrate its advantages. Previous work[12] has demonstrated that the performance of NF deteriorates when modeling multi-modal distributions separated by increasingly high energy barriers. Here, we adapt the two-dimensional analytical distributions consisting of two Gaussian modes introduced in Ref.[12] as our target distributions, defined as:

$$p(\mathbf{x}) = \frac{1}{4}\mathcal{N}(\boldsymbol{\Psi}-\boldsymbol{\Psi_1},\boldsymbol{\Sigma}_1) + \frac{3}{4}\mathcal{N}(\boldsymbol{\Psi}-\boldsymbol{\Psi_2},\boldsymbol{\Sigma}_2) \tag{21}$$

where $\boldsymbol{\Psi}\in\mathbb{R}^2$, $\boldsymbol{\Psi_1} = (-m,m)$, $\boldsymbol{\Psi_2} = (m,m)$, $\boldsymbol{\Sigma}_1 = \left(\begin{smallmatrix}0.05 & -0.035\\ -0.035 & 0.05\end{smallmatrix}\right)$ and $\boldsymbol{\Sigma}_2 = \left(\begin{smallmatrix}0.2 & -0.035\\ -0.035 & 0.2\end{smallmatrix}\right)$. The parameter m determines the positions of the two modes and, consequently, the relative barrier between them. It is varied over the set $\{1.00, 1.42, 1.84, 2.26\}$, with larger values corresponding to greater separation between the modes and higher barriers. For each target distribution, we train five RealNVP models with different random seeds using either a standard Gaussian prior (i.e., $\tau = 0$) or a tilted Gaussian prior (i.e., $\tau = 1$). Each RealNVP model is composed of 20 affine coupling layers, where the scaling and translation networks are multilayer perceptrons with two hidden layers of width 12, connected by LeakyReLU activation functions. The models are trained using 2×10^5 samples

drawn from each target distribution, with a batch size of 512. The optimization is performed using Adam with a learning rate of 5×10^{-5} for 300 epochs.

To evaluate models' performance, we quantify the average log-likelihood of samples generated by different trained models against the analytical target distributions (Figure S1(a)). Overall, we observe that as the barrier between the two Gaussian modes increases, the RealNVP models generally become less expressive in approximating the target distributions because of the increased nonlinearity, which is consistent with previous study[12]. Nevertheless, the model using the tilted Gaussian prior outperforms those using the standard Gaussian prior, as evidenced by its higher log-likelihood values and lower variance. Further visualization of the generated sample distributions (Figure S1(b)) shows that multi-modal distributions separated by higher barriers cause the NF model to more easily suffer from mode collapse, whereas the tilted Gaussian prior helps mitigate this issue to some extent. Consequently, we expect that the tilted Gaussian prior has the potential to serve as a more effective prior for generative models. In future work, more systematic tests incorporating different combinations of model architectures and training hyperparameters, including the tilting parameter τ, could be conducted across additional systems to enable more general validations and conclusions.

When training generative models on data collected at different temperatures, the prior distribution should be designed to reflect temperature dependence. A common approach, supported by prior studies, is to use a standard Gaussian prior with variance scaled proportionally to the temperature, which corresponds to the equilibrium distribution of a simple harmonic oscillator at that temperature[13–15]. Here, we propose a more generalized and flexible temperature-dependent tilted Gaussian prior, in which the distribution's variance and mode (most probable radius) vary with temperature. This design yields a more informative prior that better captures temperature-dependent behaviors and more accurately distinguishes differences between temperatures. Specifically, the probability density function at temperature T, denoted as $r_T(\boldsymbol{z}, \tau)$, is defined as follows:

$$r_T(\boldsymbol{z}, \tau) = \frac{\exp(\tau||\boldsymbol{z}||)}{Z_{\tau,T}} \cdot \frac{\exp(-\frac{1}{2T}||\boldsymbol{z}||^2)}{(2\pi)^{\frac{d_z}{2}}(T)^{\frac{d_z}{2}}} \tag{22}$$

$$= \frac{1}{Z_{\tau,T}\ (2\pi)^{\frac{d_z}{2}}(T)^{\frac{d_z}{2}}} \exp(-\frac{1}{2T}(||\boldsymbol{z}|| - T\tau)^2 \exp(\frac{1}{2}\tau^2 T^2). \tag{23}$$

Completing the square shows that the temperature-dependent tilted Gaussian is radially symmetric, with both the radius of maximum probability and variance varying linearly with temperature. $Z_{\tau,T}$ denotes the temperature-dependent normalization factor, which could be analytically derived:

$$Z_{\tau,T} = \int_{\mathbb{R}^{d_z}} e^{\tau||\boldsymbol{z}||}\ \frac{\exp(-\frac{1}{2T}||\boldsymbol{z}||^2)}{(2\pi)^{d_z/2}\ (T)^{d_z/2}}\ d\boldsymbol{z} \tag{24}$$

$$= \int_0^{+\infty} S_{dz}(r) \cdot e^{\tau r} \cdot \exp(-\frac{1}{2T}r^2)/(2\pi)^{d_z/2}(T)^{d_z/2}\ dr \tag{25}$$

$$= \int_0^{+\infty} \frac{(2\pi)^{d_z/2}}{\Gamma(\frac{d_z}{2})} r^{n-1} \cdot e^{\tau r} \cdot \exp(-\frac{1}{2T}r^2)/(2\pi)^{d_z/2}(T)^{d_z/2}\ dr \tag{26}$$

$$= M(\frac{d}{2}, \frac{1}{2}, \frac{T}{2}\tau^2) + \tau\sqrt{2T}\frac{\Gamma(\frac{d+1}{2})}{\Gamma(\frac{d}{2})} M(\frac{d+1}{2}, \frac{3}{2}, \frac{T}{2}\tau^2). \tag{27}$$

III. NORMALIZING FLOWS AND REAL NVP TRANSFORMATIONS

Probabilistic generative models seek to approximate a target probability distribution from a finite number of samples and quickly generate new samples obeying this estimated density. NF constitute one representative class of probabilistic generative models and generally use a series of invertible transformations to bridge a simple prior distribution to an approximation of the target distribution. NF models are trained directly to maximize the likelihood of observing the empirical samples given a set of learnable functions connecting the prior distribution and target distribution. These functions can then rapidly transform samples drawn from the prior distribution to the target distribution.

Specifically, NF models seek to learn an invertible function $\mathcal{F}$ that transforms a sample $\boldsymbol{x}$ from the target distribution $p_{\boldsymbol{x}}$ into a sample $\boldsymbol{z}$ obeying a simple prior distribution $p_{\boldsymbol{z}}$ via $\boldsymbol{z} = \mathcal{F}(\boldsymbol{x})$. Before considering optimization, we note the change in probability due to a change in coordinates $\boldsymbol{z} = \mathcal{F}_\theta(\boldsymbol{x})$ (θ indicates a parameterization of $\mathcal{F}$) is:

$$p_{\boldsymbol{x}}(\boldsymbol{x};\theta) = p_{\boldsymbol{z}}(\mathcal{F}_\theta(\boldsymbol{x})) \left| \det\left(\frac{\partial \mathcal{F}_\theta(\boldsymbol{x})}{\partial \boldsymbol{x}^\top}\right)\right| \tag{28}$$

where the Jacobian determinant of the transformation shows up on the right-hand side. Taking the logarithm of both sides yields:

$$\log p_{\boldsymbol{x}}(\boldsymbol{x};\theta) = \log p_{\boldsymbol{z}}(\mathcal{F}_\theta(\boldsymbol{x})) + \log \left| \det\left(\frac{\partial \mathcal{F}_\theta(\boldsymbol{x})}{\partial \boldsymbol{x}^\top}\right)\right|. \tag{29}$$

The above formulation defines a maximum likelihood estimation problem over samples from $p_{\boldsymbol{x}}$. Given a user-defined prior distribution $p_{\boldsymbol{z}}$, and assuming the log-determinant of the Jacobian can be evaluated, one can fully characterize $p_{\boldsymbol{x}}(\boldsymbol{x};\theta)$ via the change-of-variables formula. This objective can be optimized if the transformation $\mathcal{F}$ is represented as a neural network. New samples can then be generated using $\tilde{\boldsymbol{x}} = \mathcal{F}_\theta^{-1}(\tilde{\boldsymbol{z}})$, where $\tilde{\boldsymbol{z}} \sim p_{\boldsymbol{z}}$. If $\mathcal{F}_\theta$ is sufficiently expressive and optimization is successful, the generated samples will follow the target distribution p_x. Meanwhile, the generated probability density can be evaluated using the Jacobian determinant.

The functional form of reversible bridging function $\mathcal{F}_\theta$ and the associated neural network architecture varies across different NF implementations. In this work, we adopt the *Real NVP*, or real-valued, non-volume preserving transformations framework [16], which balances simplicity and expressiveness. In Real NVP, $\mathcal{F}_\theta$ is constructed as a sequence of discrete, invertible transformations known as *coupling layers*. These layers are designed such that the Jacobian of the overall transformation is triangular, allowing the determinant to be computed efficiently as a simple product of diagonal terms, without cross dependencies. The tradeoff between transformation speed and model expressivity when choosing the functional form of the transformations is a general challenge that various NF implementations approach differently [17, 18].

The Real NVP NF is a composition of n coupling layers:

$$\boldsymbol{z} = \mathcal{F}_\theta(\boldsymbol{x}) = (\boldsymbol{f}_\theta^n \circ \boldsymbol{f}_\theta^{n-1} \circ \cdots \circ \boldsymbol{f}_\theta^1)(\boldsymbol{x}) \tag{30}$$

An example of one coupling layer $\boldsymbol{y} = \boldsymbol{f}_\theta(\boldsymbol{w})$ is as follows, where $\boldsymbol{w}_{1:D}$ is an input vector and $\boldsymbol{y}_{1:D}$ is an output vector, both with D components (note that the input $\boldsymbol{w}$ will necessarily only correspond to the actual sample $\boldsymbol{x}$ at the first coupling layer $\boldsymbol{f}_\theta^1$):

$$\boldsymbol{y}_{1:d} = \boldsymbol{w}_{1:d} \tag{31}$$

$$\boldsymbol{y}_{d+1:D} = \boldsymbol{w}_{d+1:D} \odot \exp(\boldsymbol{S}_\theta(\boldsymbol{w}_{1:d})) + \boldsymbol{T}_\theta(\boldsymbol{w}_{1:d}) \tag{32}$$

where $\odot$ is the element-wise multiplication operation and the multi-valued scaling function $\boldsymbol{S}_\theta$ and translation function $\boldsymbol{T}_\theta$ are parameterized by neural networks. For layer-wise likelihood maximization, we require the determinant of the following matrix:

$$\frac{\partial \boldsymbol{f}_\theta}{\partial \boldsymbol{w}^\top} = \frac{\partial \boldsymbol{y}}{\partial \boldsymbol{w}^\top} = \begin{bmatrix} \mathbf{I}_{d\times d} & \mathbf{0} \\ \frac{\partial \boldsymbol{y}_{d+1:D}}{\partial \boldsymbol{w}_{1:d}^\top} & \mathrm{diag}(\exp \boldsymbol{S}_\theta(\boldsymbol{w}_{1:d})) \end{bmatrix} \tag{33}$$

These coupling layers alternate so that after each one, only a subset $\{d+1 : D\}$ of the components of the input vector have changed. We can then switch the order in the next layer so that the components with indices $\{d+1 : D\}$ stay unchanged, while $\{1 : d\}$ are transformed. The Jacobians of the transformations are either upper- or lower-triangular, so the determinant is simply the product along the main diagonal. The overall Jacobian determinant reduces to the product of the determinants of the individual layers, allowing one to write the negative log-likelihood of one data sample $\boldsymbol{x}$ under a given set of model parameters as:

$$\mathcal{L} = -\log p_{\boldsymbol{x}}(\boldsymbol{x};\theta) = -\log p_{\boldsymbol{z}}(\mathcal{F}_\theta(\boldsymbol{x})) - \sum_{k=1}^{n} \log \left| \exp \sum_i (\boldsymbol{S}^k_{\theta,i}(\boldsymbol{w}^k_{1:d})) \right| \tag{34}$$

where $\boldsymbol{w}^k_{1:d}$ is the input to $\boldsymbol{S}^k_\theta$ at coupling layer k, and i indexes the components of $\boldsymbol{S}^l_\theta$. We can easily extend this single-sample loss function to batches by summing the losses due to the additive property of the log-likelihood. The simple yet powerful Real NVP architecture can be made more expressive by increasing the number of coupling layers or the depth of the neural networks at each layer. While we find Real NVP adequate for our purposes, other attractive architectures, such as GLOW [19] and Neural Spline Flows [20], approach balancing expressivity and evaluation speed differently. GLOW was tested as a candidate for integration with SPIB, but its channel-wise convolutions proved less suitable for the low-dimensional latent data of this study than for its intended image domain.

IV. DETAILS OF MOLECULAR DYNAMICS SIMULATIONS

A. Three-hole Potential System

The analytical expression for the 2-dimensional three-hole potential is given by:

$$V(x,y) = 3e^{-x^2-(y-\frac{1}{3})^2} - 3e^{-x^2-(y-\frac{5}{3})^2} - 5e^{-(x-1)^2-y^2} - 5e^{-(x+1)^2-y^2} + 0.2x^4 + 0.2(y-\frac{1}{3})^4 \quad (35)$$

We perform MD simulation of a single particle with mass $m = 1$ on this three-hole potential energy surface. The dynamics are propagated using the Langevin middle integrator[21] with a time step of 0.001. The system temperature is maintained at $1/k_B$ via a Langevin thermostat employing a friction coefficient of 0.5 $step^{-1}$. To confine the particle within the 2-dimensional simulation domain, reflective boundary conditions are applied, restricting the particle to the range $x \in [-2.3, 2.3]$, $y \in [-1.7, 2.6]$. The simulation is performed for a total of 5×10^7 integration steps, with particle coordinates recorded every 50 steps, resulting in a trajectory consisting of 10^6 frames.

B. Chignolin Protein

Chignolin (GYDPETGTWG) is a 10-residue β-hairpin miniprotein, originally engineered by Honda et al.[22] based on a consensus sequence derived from turn motifs structurally analogous to those found in the GB1 hairpin domain. The NMR-resolved structure of Chignolin (PDB ID: 1UAO)[22] is employed as the initial conformation for our MD simulations. The protein is first solvated in a dodecahedral simulation box containing 2,300 explicit TIP3P water molecules[23] and then neutralized with 100 mM NaCl. The box dimensions are selected to ensure that protein residue is positioned at least 1.3 nm away from the box boundaries, and periodic boundary conditions are applied along all dimensions. The OPLS-AA force field[24, 25] is utilized for the protein, as it has recently been benchmarked to deliver the most reliable performance for this system[26]. In the simulations, electrostatic interactions are truncated at a distance of 1.2 nm, with long-range contributions treated using the Particle Mesh Ewald (PME) method[27]. Van der Waals interactions are similarly computed with a cutoff of 1.2 nm. All simulations are performed using a 2 fs integration time step, with all covalent bonds involving hydrogen atoms constrained using the LINCS algorithm[28].

To evaluate the generative capability of the LaTF model across a range of temperatures, we conduct six independent ultra-long MD simulations at 340 K, 360 K, 380 K, 400 K, 420 K, and 440 K, respectively. Prior to the production runs, the system is first subjected to energy minimization for 5,000 steps using the steepest descent algorithm, followed by a multi-stage equilibration protocol at different target temperatures. The equilibration protocol consists of five sequential phases: (i) a 2 ns NVT equilibration in which all protein atoms are restrained to their energy-minimized positions using a harmonic potential with a force constant of 1000 $kJ/(mol \cdot nm^2)$; (ii) a 2 ns NPT equilibration using Berendsen pressure coupling[29] with the same positional restraints; (iii) a subsequent 2 ns NPT equilibration with a reduced restraint force constant of 100 $kJ/(mol \cdot nm^2)$; (iv) another 2 ns NPT equilibration with a further reduced force constant of 10 $kJ/(mol \cdot nm^2)$; and finally, (v) a 2 ns NPT equilibration using Parrinello–Rahman pressure coupling[30] with all restraints removed. After energy minimization and equilibration, NVT production simulations are performed from the equilibrated configurations at six distinct temperatures. The coordinates of the Chignolin protein are recorded every 0.5 ns, and the trajectory lengths corresponding to each temperature are listed in Table S1. We observe that each trajectory exhibits more than 20 reversible folding–unfolding transitions, indicating that all simulations effectively sample the global energy landscape of the Chignolin protein. All Chignolin protein simulations are performed with GROMACS[31].

Table S1: Summary of molecular dynamics trajectory lengths for Chignolin simulated at different temperatures.

Temperature	340K	360K	380K	400K	420K	440K
Trajectory Length (μs)	35.04	35.25	34.98	17.39	35.63	35.66

C. Lennard-Jones 7 System

The Lennard-Jones 7 (LJ7) system is a widely used model for studying colloidal rearrangements[32–34]. It comprises seven particles confined to a 2-dimensional space, interacting via the Lennard-Jones potential. The LJ7 system exhibits pronounced temperature-dependent behavior, characterized by a marked disparity between the configurational spaces sampled at low and high temperatures. Therefore, this model may serve as an effective benchmark for evaluating the performance of LaTF, particularly in its ability to accurately capture key temperature-dependent behaviors from limited data. We perform six independent MD simulations of the LJ7 system across a range of temperatures, from $0.2\epsilon/k_B$ to $0.7\epsilon/k_B$, in increments of $0.1\epsilon/k_B$. Specifically, the integration time step is set to $0.005\sqrt{m\sigma^2/\epsilon}$, and a Langevin thermostat with a friction coefficient of $0.1\sqrt{\epsilon/m\sigma^2}$ is employed to maintain constant temperature throughout each simulation. The cutoff distances for force calculations and neighbor list construction are chosen as 2.5σ and 3.0σ, respectively. For each temperature, we perform a simulation of 10^7 steps and record the coordinates of all particles every 100 steps, resulting in a trajectory comprising 10^5 snapshots. All LJ7 simulations are performed with PLUMED[35].

D. GCAA RNA Tetraloops

The hyperstable GCAA tetraloop (ggcGCAAgcc) has been widely used as a benchmark system for evaluating advances in RNA force fields and sampling methodologies. We first generate an ensemble of diverse structures using RNA structure prediction tools to comprehensively sample its rugged energy landscape. Specifically, the thermodynamics-based secondary structure prediction tool ViennaRNA [36, 37] is employed to predict both the most stable and suboptimal secondary structures of the RNA using sequence information alone, resulting in three different secondary structures. The Fragment Assembly of RNA with Full Atom Refinement (FARFAR) method in Rosetta[38–40] is employed to sample corresponding tertiary structures using Monte Carlo sampling combined with a knowledge-based scoring function, resulting in 3,000 tertiary conformations. Subsequently, all 3,000 structures are characterized using G-vector descriptors [41], projected onto the top four principal components via Principal Component Analysis (PCA), and grouped into 20 clusters using the K-means algorithm. The structures closest to the geometric center of each cluster are selected as the initial seeds for MD simulations.

We perform independent, unbiased MD simulations at 300 K and 400 K, respectively. The simulation system is constructed with a dodecahedral box containing the initial RNA structure, 3,900 water molecules, and is neutralized with 1 M KCl. The box size is chosen to ensure all RNA atoms are positioned at least 1.3 nm from the box boundaries, and periodic boundary conditions are applied across every dimension. To model the GCAA tetraloop, we employ the recently developed classical RNA force field DESRES-AMBER[42], an enhanced extension of the AMBER ff14 force field[43], which has been benchmarked through extensive simulations across various RNA systems[42, 44]. Following the recommended protocol, we use the TIP4P-D water model[45] and apply the CHARMM22 ion parameters[46]. The cutoff for Coulombic interactions and van der Waals interactions is consistently set to 1.2 nm, with long-range electrostatics computed using the PME method[27]. The integration time step is chosen as 2 fs, and all bonds involving hydrogen atoms are constrained using the LINCS algorithm[28]. Following the same equilibration protocol used in the Chignolin protein simulations described above, we perform energy minimization, a 2 ns NVT relaxation with positional restraints, three successive 2 ns NPT relaxations with gradually reduced restraint forces, and a final 2 ns NPT relaxation without restraints. Production NVT simulations are then initiated from the equilibrated configurations, and RNA coordinates are recorded every 1.0 ns.

The interpolation and generative capabilities of the LaTF model offer a powerful tool for reseeding simulations and conducting adaptive sampling. Specifically, we conduct four iterations of adaptive sampling, each comprising 20 independent simulations. Between each iteration, the LaTF model is trained on the accumulated data collected so far at 300 K and 400 K, using an appropriate lag time to enable the identification of 10 metastable states in the latent space. For the next iteration, we select the lowest free energy configuration from each state and midpoints interpolated between each pair of nearest low–free-energy configurations as starting points for new simulations (see details in Sec. V (D)). A total of 80 trajectories at 300 K and 80 trajectories at 400 K were obtained, with average lengths of $\sim 2.37\mu s$ and $\sim 1.85\mu s$, respectively; the distributions of trajectory lengths are shown in Figure S9 and S10.

Given the inherently rugged and complex energy landscape of RNA, it is challenging to directly infer a converged

landscape from parallel short simulations, particularly at lower temperatures. To ensure that the data used to train the LaTF model is thermodynamically and kinetically unbiased, we generate long-timescale trajectories at two temperatures using Markov State Models (MSMs) for training. For each temperature, we first represent tetraloop structures from MD simulations using $\boldsymbol{r}$-vectors and carbon atom pairwise distances (see Sec. V(B)), and project them into a 5-dimensional kinetic space using Time-lagged Independent Component Analysis (TICA)[47] with kinetic mapping [48]. The projected conformations are then clustered into 200 microstates using the K-means algorithm, and MSM is constructed with a 200 ns lag time. While MSM hyperparameters could be further optimized using cross-validation with the generalized matrix Rayleigh quotient (GMRQ)[49] or Variational approach to Markov Processes (VAMP) score[50], we adopt this setup to focus on generating ultra-long unbiased trajectories and validate the models using Implied Timescale (ITS) analysis and the Chapman-Kolmogorov (CK) test[51] (see Figure S9 and S10). When using the LaTF model to infer the melting curve of the tetraloop, we define folded conformations as those in which all three Watson–Crick base pairs are formed and the all-atom RMSD to any NMR structure is below 4.0 Å. Watson–Crick interactions are identified using the Barnaba Python package[52], which incorporates both distance- and orientation-based metrics. We evaluate the sensitivity of the RMSD threshold and find that, across a range of 3.6–4.4 Å, the predicted melting curves remain consistent and in good agreement with both experimental measurements and simulated tempering results.

V. TRAINING OF LATENT THERMODYNAMIC FLOWS

A. Feature Extraction and Initialization of State Labels

Three-hole Potential System

The (x, y) coordinates are provided as input to LaTF, and the initial labels are obtained by applying K-means clustering to the trajectories in the (x, y) space, resulting in 100 clusters.

Chignolin Protein

Chignolin protein structures are featurized using pairwise distances between carbon-alpha atoms of residue pairs that are separated by at least two positions along the sequence, resulting in a 28-dimensional feature vector. For the single-temperature trainings at 340 K, initial labels are generated using TICA[47] with kinetic mapping[48] followed by K-means clustering, which has proven effective for SPIB training of protein systems[2]. Specifically, three independent components are selected based on spectral gap analysis[53], and the projected data are partitioned into 100 clusters. For training with data from two temperatures, initial labels are generated using PCA followed by K-means clustering. Feature vectors from both temperatures are concatenated and projected onto the top 10 principal components, ensuring that any remaining component contributes less than 1% to the explained variance ratio. The resulting projected structures are then clustered into 100 clusters.

Lennard-Jones 7 System

The 2-dimensional configurations of the seven-particle system are described using coordination numbers. Specifically, for each particle i, the coordination number descriptor c_i is defined as:

$$c_i = \sum_{j=1, j\neq i}^{7} s_{ij} \tag{36}$$

where s_{ij} represents the switching function applied to the pairwise contact distance r_{ij} between particles i and j, defined as:

$$s_{ij} = \frac{1 - (r_{ij}/r_0)^8}{1 - (r_{ij}/r_0)^{16}} \quad (r_0 = 1.5\sigma) \tag{37}$$

The switching function ensures that the coordination number is a smooth, continuous function with well-defined derivatives. To ensure that the structural embedding is invariant to particle permutations, the concatenated coordination number for each snapshot is sorted before being input into the LaTF model.

Previous studies[32, 34] have shown that the second and third moments of the coordination numbers, $\mu_2^2 = \frac{1}{7}\sum_{i=1}^{7}(c_i - \langle c_i \rangle)^2$ and $\mu_3^3 = \frac{1}{7}\sum_{i=1}^{7}(c_i - \langle c_i \rangle)^3$, serve as informative and meaningful order parameters (OPs) for the LJ7 system. Initial labels for LaTF training are generated using these two empirical OPs. Specifically, trajectories collected at two different temperatures are projected onto μ_2^2 and μ_3^3 and subsequently clustered into 100 clusters via the K-means algorithm.

GCAA RNA Tetraloops

The RNA structures are represented using two internal descriptors: $\boldsymbol{r}$-vectors[41] and pairwise distances between ribose C_4' carbon atoms. The $\boldsymbol{r}$-vectors are constructed by concatenating the relative position vectors between the centers of the six-membered rings for all nucleotide pairs. Noting that the distance information captured by $\boldsymbol{r}$-vectors effectively characterizes and distinguishes various interaction patterns such as base stacking and Watson–Crick or non–Watson–Crick base pairing[41], we therefore use only the norms of the $\boldsymbol{r}$-vectors as structural descriptors. As a result, each GCAA RNA structure is represented as a 90-dimensional feature vector, which is further used as input for LaTF training. By concatenating the featurized simulation data from 300 K and 400 K, we apply PCA to project all RNA configurations onto the top 12 principal components, such that

the explained variance of the remaining components is below 1%. The projected data are then grouped into 100 clusters using the K-means algorithm, yielding the initial labels for LaTF training.

B. Training Procedure

The training procedure of LaTF majorly mirrors that of SPIB[2], except that the on-the-fly update of the variational mixture of posteriors prior is omitted, as these are replaced by a more expressive normalizing flow architecture. LaTF is also trained in a self-consistent and iterative manner, where the number of states and their corresponding labels are dynamically updated to maximize state metastability. The pseudocode for LaTF training is provided in Algorithm 1.

In practice, we observe that when the number of initial states is relatively large, the training process of the whole LaTF model requires a significantly long time to reach loss convergence during the first few refinements. An effective strategy to accelerate the training process is to first pretrain the SPIB part (i.e., the encoder and decoder) using the vanilla SPIB objective and protocol to obtain a reasonably converged set of metastable states. Afterward, the normalizing flow component is incorporated, and all modules are trained jointly with the full LaTF objective function. We note the results obtained from these two training procedures are found to be highly consistent across all systems examined in this study.

Algorithm 1 Latent Thermodynamic Flows

Input: MD simulation trajectories at one or multiple temperatures $\{\mathbf{X}^i_{(T_i)}\}_{i=1}^L$, corresponding set of initial state labels $\{\mathbf{y}^i_{(T_i)}\}_{i=1}^L$, encoder $\mathbb{E}_\theta$ and decoder $\mathbb{D}_\theta$ networks, normalizing flow model $\mathcal{F}_\theta$ with a specified number of coupling layers, lag time Δt, convergence threshold thd, convergence patience n_{patience} for stopping criteria, number of refinement iterations $n_{\text{refinements}}$, tilted Gaussian prior parameter τ

```
Preprocessing: create training dataset such that each sample consists of {X^i_(T_i), y^{i+Δt}_(T_i), T_i}
Set loss_0, loss_1 ← 0
for iter from 1 to n_refinements do
    repeat
        Set loss_0 = loss_1
        Sample a batch of training data {X^i_(T_i)}, {y^{i+Δt}_(T_i)} and {T_i}
        loss_1 ← calculate the objective function L_LaTF, which includes the prediction accuracy from {X^i_(T_i)} to future
    state {y^{i+Δt}_(T_i)}, and a regularization term enforcing the mapping of latent representations to {T_i, τ} temperature-steerable
    tilted Gaussian priors via the normalizing flow
        Update the parameters θ of neural networks including the encoder, decoder and normalizing flow
    until the condition |loss_0 − loss_1| < thd holds for n_patience consecutive updates
    Update the state labels {y_i^(T_i)} via ŷ^i_(T_i) = argmax_i D_i(E_θ(X^i_(T_i)); Δt, θ)
end for
```

C. Network Architecture and Training Hyperparameters

To optimize training efficiency, we recommend a two-stage training protocol for the LaTF model. First, pretrain the vanilla SPIB using its original loss function to obtain a reasonably converged metastable state classification. Then, jointly train the encoder, decoder, and normalizing flow using the LaTF objective. All hyperparameters used in the two-stage training protocol are listed in Table S2 and Table S3.

Two critical hyperparameters in LaTF are the dimension of the latent space and the tilting factor τ. As demonstrated in prior studies[2], unlike dimensionality reduction methods based on the VAMP theory [50], which extract orthogonal slow modes and thus can only separate a limited number of metastable states per component, SPIB can accommodate a substantially larger number of metastable states even within a 2-dimensional IB space[2]. Accordingly, all results presented in this manuscript are consistently obtained using a 2-dimensional IB space for LaTF. Meanwhile, the choice of the tilting prior factor τ could effect the generation accuracy. For the single-temperature training case, we perform cross-validation by training LaTF models across a range of τ values (the entire dataset is divided into five folds, with four used for training and one reserved for validation), and then select the value that

Table S2: Neural network architectures and training hyperparameters used for pretraining the State Predictive Information Bottleneck (SPIB) across the different systems presented in this study.

**System**	SPIB Parameters								
	Learning Rate	β Weight	Encoder Neurons	Decoder Neurons	Lagtime Δt	Batch Size	Convergence Threshold	Convergence Patience	Refinement Number
Three-Hole Potential ($k_BT = 1.0$)	10^{-3}	10^{-4}	16	16	1500 steps	512	10^{-3}	3	10
Chignolin Protein (340K)	10^{-3}	3×10^{-4}	32	32	$30ns$	512	10^{-3}	3	15
Chignolin Protein (340K & 440K)	10^{-3}	10^{-4}	32	32	$5ns$	512	10^{-3}	5	15
Chignolin Protein ($1\mu s$ traj, 340K & 440K)	10^{-2}	5×10^{-4}	32	32	$5ns$	256	10^{-3}	10	15
Chignolin Protein (380K & 440K)	10^{-3}	10^{-4}	32	32	$5ns$	512	10^{-3}	5	15
Lennard-Jones 7 ($k_BT = 0.2\epsilon$ & 0.5ϵ)	2×10^{-3}	10^{-4}	16	16	100 steps	512	10^{-3}	5	15
Lennard-Jones 7 ($k_BT = 0.2\epsilon$ & 0.7ϵ)	2×10^{-3}	10^{-4}	16	16	100 steps	512	10^{-3}	5	15
CGAA RNA (300K & 400K)	2×10^{-3}	5×10^{-5}	32	32	$200ns$	512	0.001	5	15

Table S3: Neural network architectures and training hyperparameters used for training the Latent Thermodynamic Flows (LaTF) across the different systems presented in this study.

**System**	Latent Thermodynamic Flows Parameters								
	Learning Rate	β Weight	Coupling Layers	τ Value	Flow Neurons	Batch Size	Convergence Threshold	Convergence Patience	Refinement Number
Three-Hole Potential ($k_BT = 1.0$)	10^{-3}	10^{-4}	10	3	16	512	0.1	200	1
Chignolin Protein (340K)	10^{-3}	3×10^{-4}	35	3	16	512	0.1	200	1
Chignolin Protein (340K & 440K)	10^{-3}	10^{-4}	35	2.5	16	512	0.1	150	0
Chignolin Protein ($1\mu s$ traj, 340K & 440K)	10^{-2}	5×10^{-4}	35	3	16	256	0.1	150	0
Chignolin Protein (380K & 440K)	10^{-3}	10^{-4}	35	3.5	16	512	0.1	150	0
Lennard-Jones 7 ($k_BT = 0.2\epsilon$ & 0.5ϵ)	2×10^{-3}	10^{-4}	20	2	16	512	0.1	150	0
Lennard-Jones 7 ($k_BT = 0.2\epsilon$ & 0.7ϵ)	2×10^{-3}	10^{-4}	20	2	16	512	0.1	150	0
CGAA RNA (300K & 400K)	2×10^{-3}	5×10^{-5}	50	4.5	16	4096	0.1	150	0

results in generated distributions with the smallest average symmetric KL divergence from the validation-set distributions. For the multi-temperature case, we adopt a similar cross-validation strategy, but determine the optimal τ by minimizing the symmetric KL divergence between the generated and validation distributions evaluated only at the training temperatures. In this case, the choice of τ requires greater care, as the selection criterion ensures that the temperature-dependent tilted Gaussian priors best accommodate and distinguish data from the different training temperatures.

Additionally, based on our training experience, we find that the β weight, which balances predictive and generative accuracy, is largely insensitive across a broad range of values from 5×10^{-5} to 1×10^{-4}. This observation is consistent with our previous work[2], where varying β did not affect SPIB's ability to identify the key metastable states or capture the dominant slow dynamical processes. In the future, if one wishes to optimize LaTF specifically for recovering the most important slow dynamics, cross-validation using the GMRQ score may be employed to guide

the selection of β.

Apart from to the KL divergence, reconstruction loss, and GMRQ evaluations presented in the main text for Chignolin, we also compare LaTF with SPIB using implied timescale (ITS) analysis and the Chapman–Kolmogorov (CK) test. Specifically, we train the SPIB model and two LaTF models with $\tau = 0$ and $\tau = 3$, respectively, using an 80%–20% cross-validation split, and construct the corresponding MSMs on the full dataset using state labels assigned by the trained models. As shown in Figure. S4(a), the LaTF models are observed to outperform the SPIB model by exhibiting faster ITS convergence, i.e., shorter Markovian lag times, and slightly larger converged timescale values, both indicative of improved metastable-state classification. In addition, the CK test results indicate that MSMs constructed using LaTF exhibit slightly better long-term predictive performance, as their predictions align more closely with results from the raw MD data (see Figure S4(b)). To enable a more systematic comparison among all models obtained from cross-validation, we introduce a scalar metric, the time-averaged root-mean-squared error (RMSE), to quantify the discrepancy between the MSM-predicted Transition Probability Matrices and those directly estimated from the MD data across different lag times:

$$RMSE = \frac{1}{6} \sum_{t=20ns}^{120ns} \sqrt{\frac{\sum_{i,j=1}^{N} \left(\pi_i T_{ij}^{MD}(t) - \pi_i T_{ij}^{MSM}(t) \right)^2}{N^2}} \tag{38}$$

where π_i denotes the stationary probability of state i, $T_{ij}(t)$ is the (i, j) element of the TPM at lag time t, and N represents the total number of metastable states. As shown in Figure S4(c), the prediction errors from SPIB-based MSMs are clearly larger than those from LaTF-based MSMs, further supporting the superior state classification capability of LaTF.

D. Choice of Information Bottleneck Dimensionality

In our current implementation, we fix the IB dimensionality at two. To further justify this choice, we evaluate the quality of the resulting 2D IB representation by comparing it with an established and deterministic CV identification method, i.e., tICA, and by assessing its ability to accommodate the metastable states identified from MSMs.

Validate Against tICA: tICA identifies independent components ranked by their associated relaxation timescales from high-dimensional descriptors. To benchmark the expressive power of LaTF's 2D IB representation, we compare it against the CVs obtained from tICA. Specifically, for both the Chignolin and RNA tetraloop systems studied in this manuscript, we conduct tICA analysis using the same structural descriptors provided to the LaTF, e.g., C_α pairwise distances for Chignolin and the $\mathbf{r}$-vector plus selected distances for the tetraloop.

To determine the effective number of tICA CVs, the 'gap' in the eigen-spectrum is typically used, since the eigenvalues of the tICA generalized eige-problem, i.e., computed from the covariance-whitened time-lagged correlation matrix, reflect the characteristic relaxation timescales of the corresponding components. As shown in Figures S11(a) and S12(a), both the eigen-spectra and cumulative kinetic variance (sum of squared eigenvalues) are visualized for the Chignolin and RNA tetraloop systems. In Chignolin, a clear separation between the 3rd and 4th eigenvalues indicates that the top three tICA CVs capture the slowest dynamics of interest, while the remaining CVs mainly capture fast relaxation processes. In the RNA tetraloop system, the eigen-spectrum is more heterogeneous, reflecting RNA's intrinsically complex and heterogeneous dynamics; nevertheless, the top five tICA CVs already account for over 65% of the kinetic variance. Therefore, we further compared the top five tICA CVs with the IB coordinates using the Pearson correlation for both systems.

Specifically, during the training of LaTF (or SPIB), a lag time should be pre-set. This parameter can slightly influence the resulting IB coordinates, so Pearson correlations are evaluated across IB coordinates obtained at different lag times. As shown in Figures S11(b) and S12(b), the 2D IB spaces effectively capture the information encoded in the multi-dimensional tICA CVs. For the Chignolin protein, the 2D IB coordinates robustly capture the information from the leading two tICA CVs and also retain some information from the third tICA CV, with correlations increasing at shorter lag times. This aligns with the intuition that shorter lag times are required for the model to resolve faster transition dynamics. For the RNA tetraloop, the 2D IB space captures substantial information from the top three tICA components and also includes contributions from the next two. Thus, for the systems studied in the manuscript, the 2D IB representation produced by LaTF indeed captures the slow

dynamical information of interest beyond that contained in only the first two tICA components, effectively covering the majority of the systems' kinetic variance.

Validate Against MSMs: A higher-resolution analysis of the system dynamics can be conducted by fine-graining tICA space and building up MSMs. Specifically, we project the simulation data onto the leading 10 tICA components for Chignolin and the leading 20 components for the RNA tetraloop (chosen to retain $\geq 98\%$ of the kinetic variance) and then apply K-means clustering to obtain 1,000 microstates in each system. Based on the constructed MSMs, we analyze the eigenvalue spectra of the transition probability matrices to locate the dominant slow dynamical modes. For Chignolin (see Figure. S11(c)), a clear gap between the 6th and 7th eigenvalues indicates the presence of six metastable (long-lived) states, i.e., states within which conformations interconvert rapidly, whereas transitions between states occur on much longer timescales[54]. For the RNA tetraloop (see Figure. S12(c)), although the spectrum is more heterogeneous, a similar gap between the 6th and 7th eigenvalues is observed, suggesting at least six metastable states (with additional shorter-lived states potentially resolvable upon further refinement). Accordingly, we apply the Perron–cluster cluster analysis plus (PCCA+) algorithm to lump the 1,000 microstates into six metastable states for each system. After assigning each molecular conformation to a metastable state, we visualize the resulting state locations within the corresponding 2D IB spaces. As shown in Figures S11(d) and S12(d), the six metastable states are distinctly accommodated and separated in both systems. These results further demonstrate the capability of LaTF to learn an informative 2D IB representation that captures multiple metastable states and separates the associated multiscale transition dynamics.

General Discussion Beyond the Cases Studied in This Manuscript: The choice of a 2D IB may not always be optimal in general. Numerous prior studies have demonstrated that the 2D IB representation derived from SPIB models could be effectively applied to wide range of tasks including enhanced sampling[55–57], Markov State Modeling[2], and weighted ensemble simulations[58], across diverse systems such as protein conformational changes[2, 59], protein-ligand interactions[3], and nucleation processes[56, 57]. Especically, in Ref.[2], we systematically evaluated SPIB on three fast-folding protein systems and found that increasing the IB dimensionality from 2 to 4 did not improve SPIB's ability to capture the slowest dynamics of interest, as quantified by the GMRQ score. The 2D IB space was sufficient to accommodate approximately ten metastable states and to distinguish multiple folding pathways in these systems. In addition, other researchers in the field have benchmarked SPIB against alternative approaches, such as state-free reversible VAMPnets (SRV), on different protein-folding datasets[60]. Their results demonstrate that MSMs constructed from the 2D IB representation significantly outperform those built on 2D SRV or 2D tICA CVs in capturing the dominant slowest dynamical modes, while achieving comparable performance to MSMs constructed from multi-dimensional tICA CVs. Taken together, these results empirically suggest that a 2D IB representation often performs robustly across a variety of systems.

We attribute the strong performance of the 2D IB representation in both LaTF and SPIB to two key factors. First, unlike many models that require reconstruction of high-dimensional structural features from a low-dimensional representation, e.g., time-lagged autoencoders, LaTF and SPIB only decodes future metastable state labels from the IB space. This greatly reduces the informational demands placed on the bottleneck. Conformations that interconvert on timescales shorter than the chosen lag time naturally collapse into the same metastable state and cluster together in IB space. Consequently, a 2D IB representation can, in principle, accommodate a large number of metastable states and encode transition dynamics that span multiple timescales. Second, the use of a multi-Gaussian prior in SPIB, and the more flexible mapping between the encoded distribution and the prior enabled by normalizing flows in LaTF allows clusters of projected conformations in the IB space to be better separated. In contrast, a simple Gaussian prior would impose strong regularization on the IB distribution, causing different metastable states to mix together.

While the robustness of a 2D IB representation in LaTF and SPIB has been demonstrated and discussed, it is important to emphasize that the IB dimensionality remains a critical hyperparameter that must be chosen with care. Incoporating a higher-dimensional IB representation generally increases the model's capacity to capture information, this comes at the cost of reduced interpretability. For broader applications in the future, the information capacity of the IB should be evaluated using independent approaches like tICA, MSMs, or density peak clustering[61], to ensure that the chosen IB dimensionality is sufficient to capture the essential slow dynamics while meaningfully separating the metastable states.

E. Interpolation of Transition Pathways

To interpolate transition pathways using LaTF, we first need to identify source and sink configurations. The normalizing flow in LaTF analytically characterizes the latent distribution, making it well-suited to locate configurations with the lowest free energy. Simultaneously, the decoder assigns any given latent samples to metastable states. Together, they enable the identification of minimum free energy configurations within each metastable state, which serve as the source and sink for pathway interpolation. After flowing the source and sink configurations to the prior space, we employ two commonly used strategies for pathway interpolation: spherical linear interpolation[62] is applied to the LaTF with a standard Gaussian prior, while linear interpolation is used for the LaTF with a tilted Gaussian prior.

Specifically, spherical linear interpolation[62] connects the source $\boldsymbol{z}^{(0)}$ and the sink $\boldsymbol{z}^{(1)}$ via:

$$\boldsymbol{z}^{(\alpha)} = \frac{\sin((1-\alpha)\theta)}{\sin(\theta)}\boldsymbol{z}^{(0)} + \frac{\sin(\alpha\theta)}{\sin(\theta)}\boldsymbol{z}^{(1)} \tag{39}$$

where $\theta = \arccos(\frac{(\boldsymbol{z}^{(0)})^T \boldsymbol{z}^{(1)}}{||\boldsymbol{z}^{(0)}||\ ||\boldsymbol{z}^{(1)}||})$ denotes the angle between source and sink, and α is chosen to uniformly partition the interval $(0, 1)$. For the tilted Gaussian prior, due to its well-structured form, we directly employ linear interpolation between the central angles and radii of the source and sink configurations. All interpolated samples are subsequently passed through the inverse normalizing flow and mapped to the latent space.

In the case study of the Chignolin protein, we benchmark the interpolated transition pathways against the kinetic pathways identified via Transition Path Theory[63, 64]. Specifically, the conformations from the 340 K MD trajectory are encoded into the 2-dimensional latent space using the trained LaTF model and subsequently grouped into 200 clusters via the K-centers algorithm. A Markov State Model is constructed with a lag time of 20 ns, selected based on implied timescale analysis and validated using the Chapman–Kolmogorov test[51]. The net flux between each pair of metastable states is computed, and the Dijkstra algorithm is applied to identify all possible transition pathways. The top five flux-carrying pathways, which collectively account for 24% of the total flux, are visualized in the main text.

The Chignolin folding system also allows us to test whether saddle points on the IB free-energy landscape correspond to isocommitted, kinetically meaningful intermediates. We apply TPT and build two independent MSMs to quantify the committor distribution in IB space: (i) a 200-state MSM obtained by K-center clustering of IB-projected conformations, and (ii) the 1000-state MSM introduced earlier, constructed by K-means clustering in the 10D tICA space. For both models, source and sink states are defined as the microstates containing the unfolded and folded structures with the highest likelihood, and committor values are computed accordingly. As shown in Figure S14, projecting each conformation and coloring it by the committor of its associated microstate reveals consistent committor patterns across the two MSMs, with the second appearing slightly fuzzy due to its IB-independent state definitions. This consistency underscores the ability of the 2D IB space to separate metastable states and encode key kinetic information. We further identify two saddle points by estimating a smooth potential of mean force via kernel density estimation and computing its spatial gradients to locate grid cells where partial derivatives change sign along orthogonal directions. Both saddle points lie in regions where the committor is approximately 0.5, marking intermediates along two distinct folding pathways and corroborating that they are isocommitted.

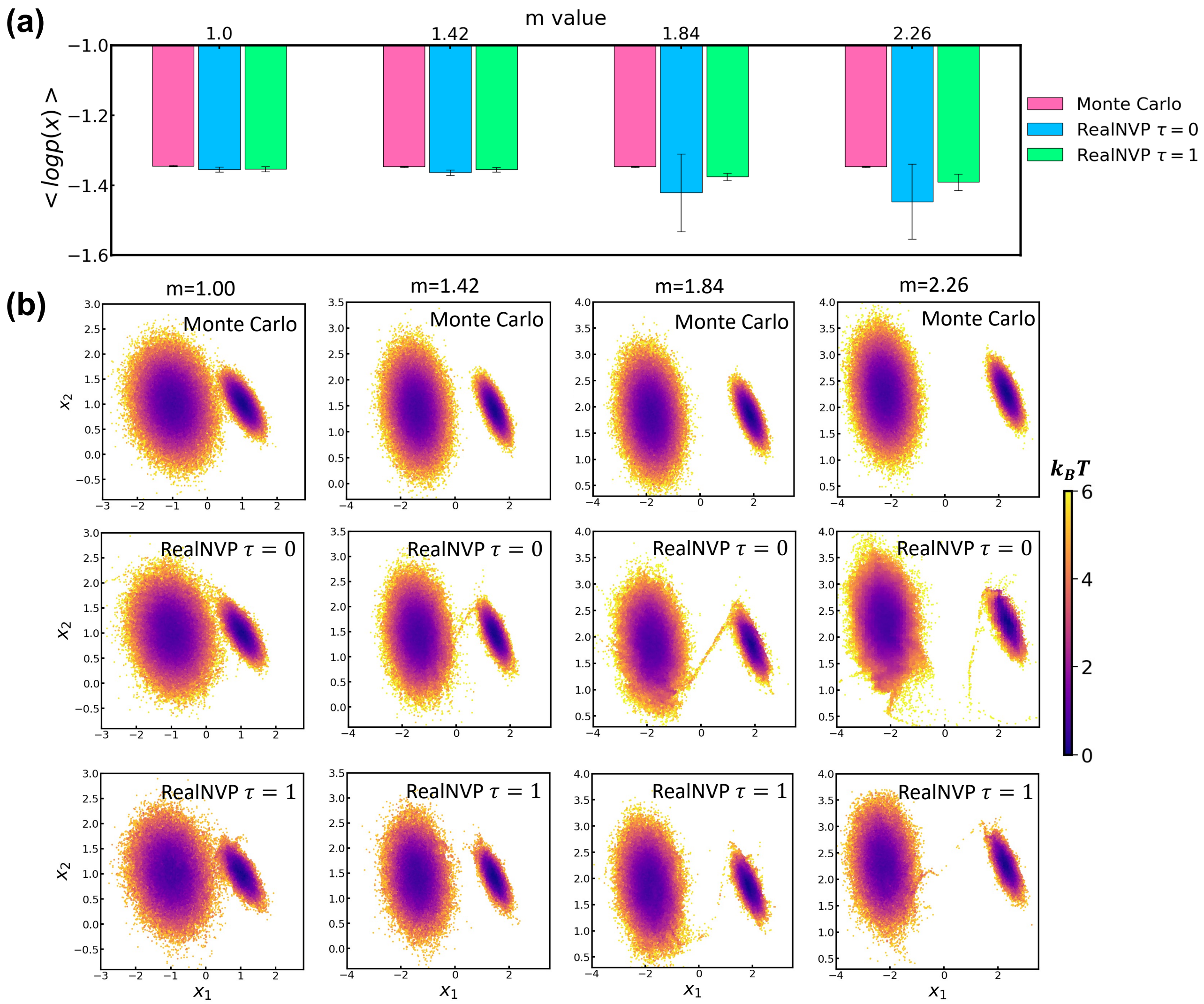

Figure S1: Performance evaluation of the Real-valued Non-Volume Preserving (RealNVP) normalizing flow (NF) with different prior structures on a two-dimensional Gaussian mixture distribution. (a) Average log-likelihood of samples generated by three methods for four target distributions with varying m, evaluated against the analytical distribution. Monte Carlo (MC) is conducted using the analytical distribution, and uncertainties are estimated from five MC runs or five NF models trained with different random seeds. (b) Examples of samples generated by different methods (rows) for four target distributions (columns). For each case, 2×10^5 samples are visualized.

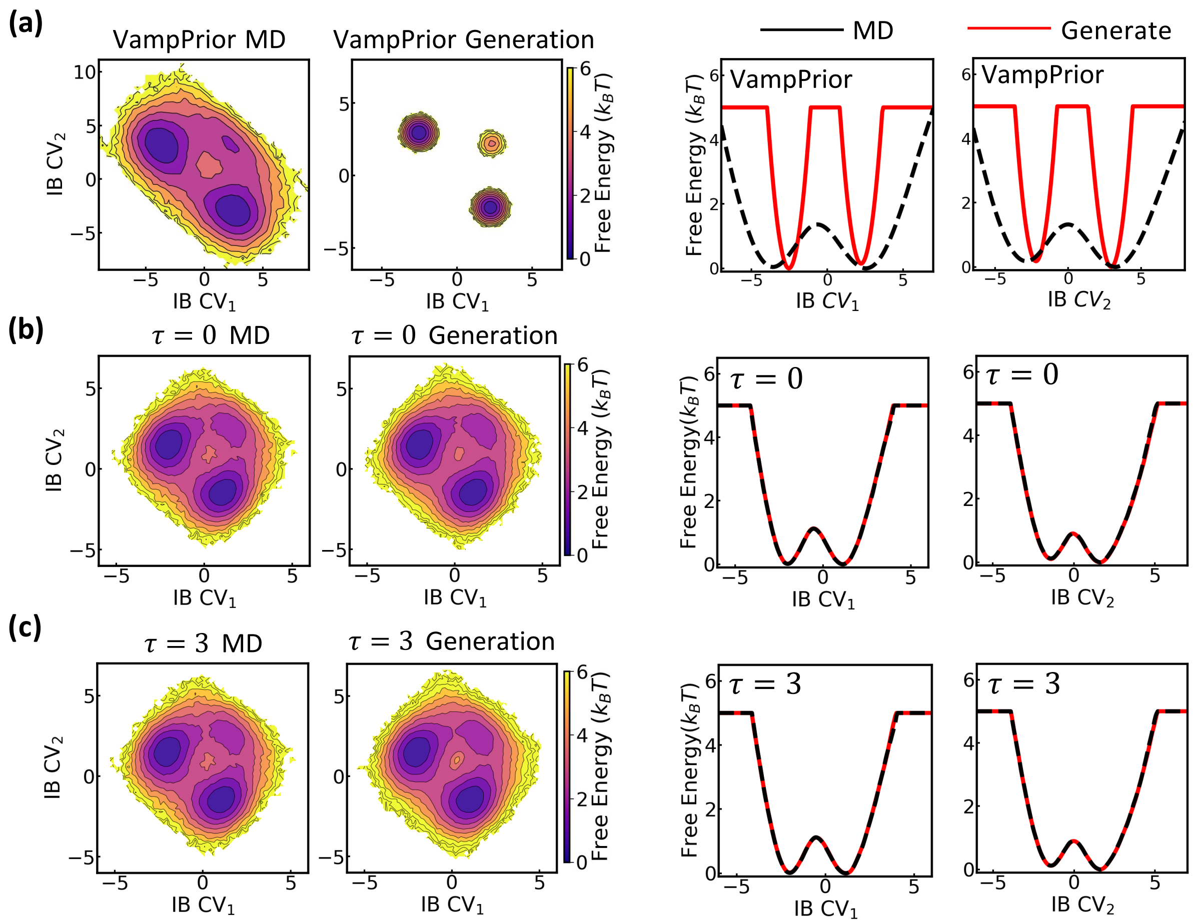

Figure S2: Comparison of generative performance between VampPrior and Latent Thermodynamic Flows (LaTF) on the 2-dimensional three-hole potential system. Visualization of the free energy landscapes in the Information Bottleneck (IB) latent space, estimated directly from encoded MD data or from samples generated by different generative models: (a) vanilla SPIB model using VampPrior; (b) LaTF model with a standard Gaussian prior; and (c) LaTF model with a tilted Gaussian prior, where the tilted parameter is set to $\tau = 3.0$.

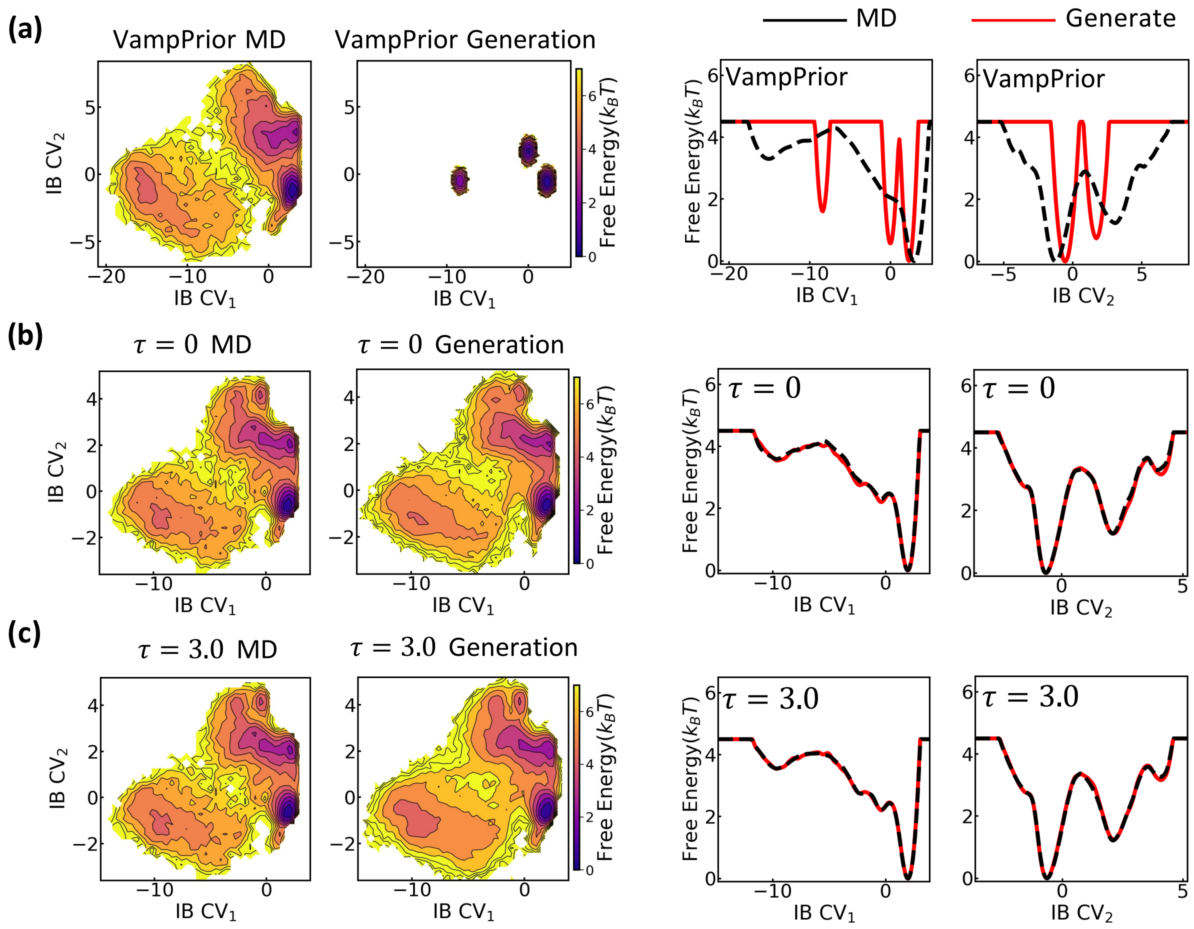

Figure S3: Comparison of generative performance between VampPrior and Latent Thermodynamic Flows (LaTF) on the Chignolin protein folding system. Vanilla State Predictive Information Bottleneck (SPIB) and Latent Thermodynamic Flows (LaTF) models with varying tilted prior parameters are trained using MD data of Chignolin at 340 K. Free energy landscapes in the encoded IB latent space, estimated either directly from MD trajectories or from samples generated by LaTF, are visualized for comparison: (a) vanilla SPIB model using VampPrior; (b) LaTF model with a standard Gaussian prior; and (c) LaTF model with a tilted Gaussian prior, where the tilted parameter is set to $\tau = 3.0$.

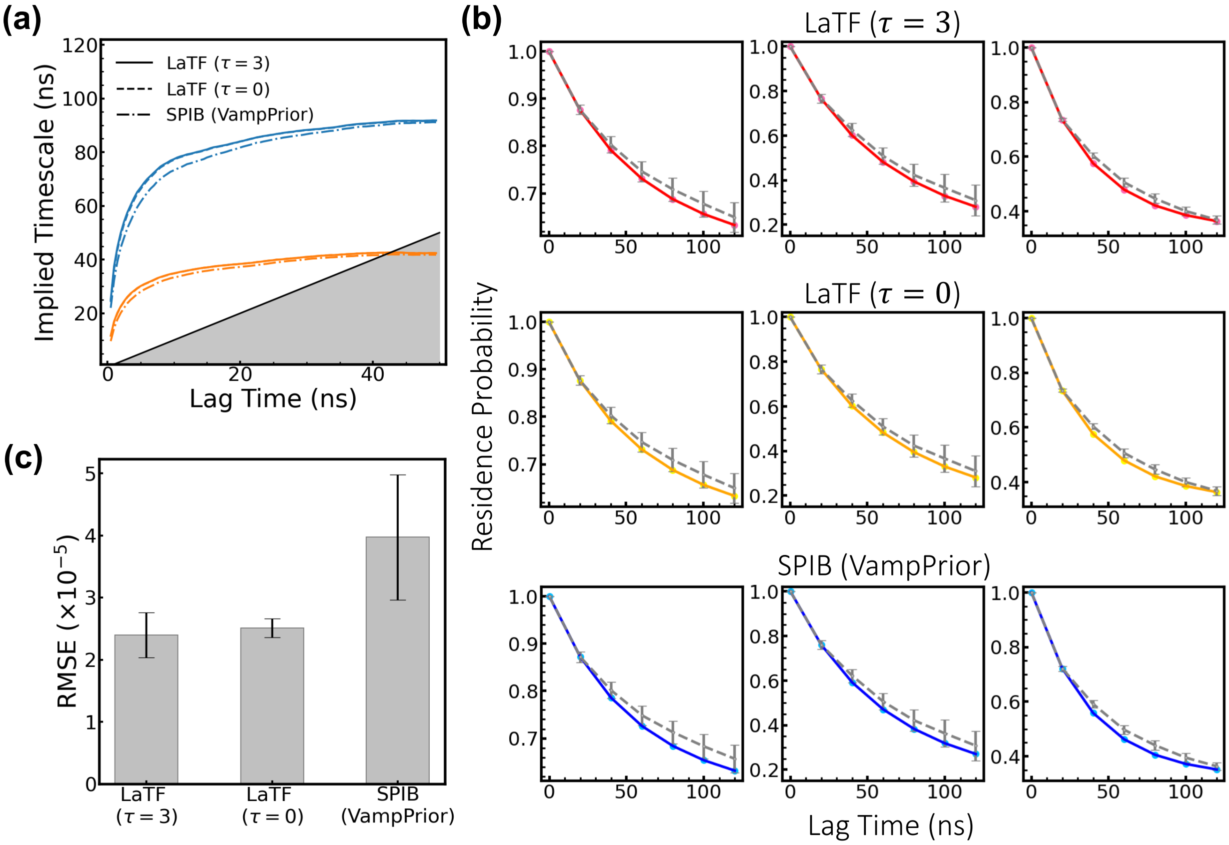

Figure S4: Evaluation of metastable-state classification quality from LaTF and SPIB using kinetic metrics for Chignolin folding at 340 K. (a) Implied timescales (ITS) of MSMs built at different lag times using metastable-state assignments obtained from LaTF with varying tilted prior parameter τ and from SPIB. Five-fold cross-validation are conducted for training of different models, and for clarity in presentation, only the mean timescale values among all the models are visualized. The shaded gray region denotes time scales that are equal to or shorter than the lag time and cannot be resolved. (b) Chapman–Kolmogorov (CK) tests for MSMs constructed with a 20 ns lag time using metastable state assignments from different models. Results from one model in the five-fold cross-validation are shown, and only the residence probabilities are reported. The grey dashed lines indicate values directly estimated from MD data, whereas the colored lines denote MSM predictions. Uncertainties are obtained by uniformly partitioning the trajectory into 20 segments and performing bootstrap resampling. (c) The time-averaged root-mean-squared error (RMSE) between MSM-predicted Transition Probability Matrices (TPMs) and TPMs directly estimated from MD data. Uncertainties are obtained from the five-fold cross-validation.

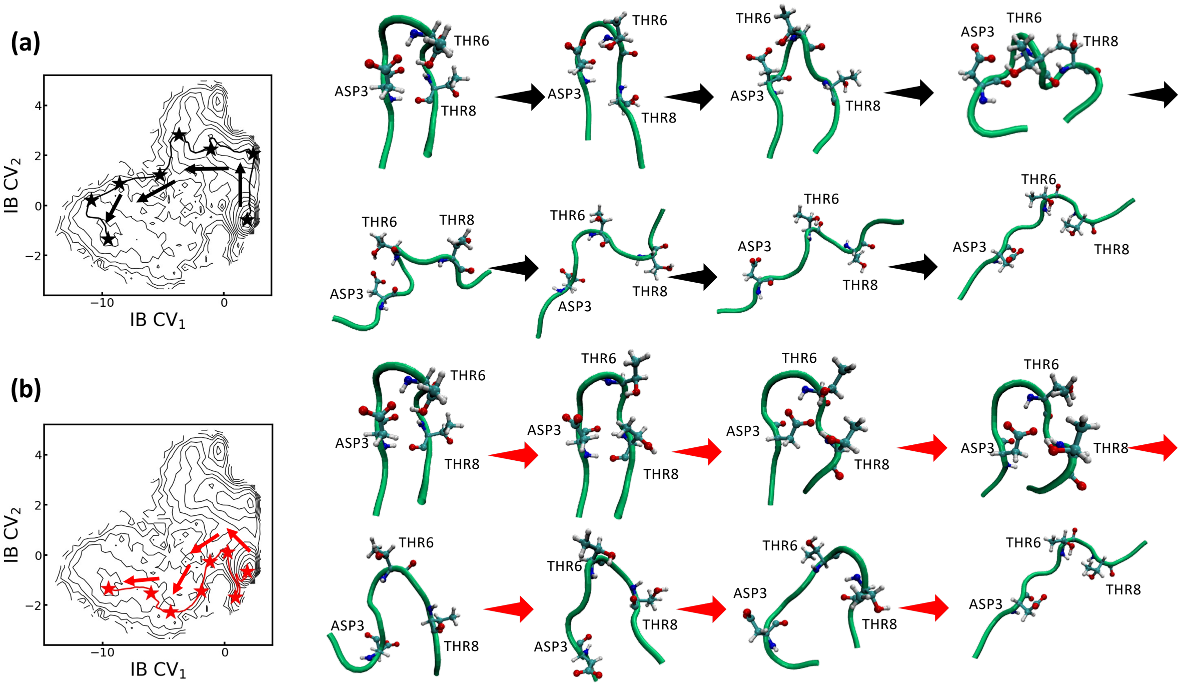

Figure S5: Two transition pathways interpolated by the Latent Thermodynamic Flows (LaTF) model for the Chignolin protein, along with key intermediate conformations identified along each pathway. The LaTF model is trained on MD data at 340 K with a tilted prior parameter of $\tau = 3$. The most probable conformations in the latent space from the folded and unfolded states are selected as the source and sink, respectively, with their probabilities quantified using the Jacobian determinant of the normalizing flow transformation. Linear interpolation is performed between the source and sink in the tilted Gaussian prior space, and the interpolated points are mapped back to the latent space. For each pathway, six generated intermediate conformations are selected and reconstructed to all-atom structures using nearest neighbors in the latent space. Three key residues that serve as markers distinguishing the two folding pathways are highlighted. The two interpolated pathways reveal distinct folding mechanisms of the Chignolin protein.

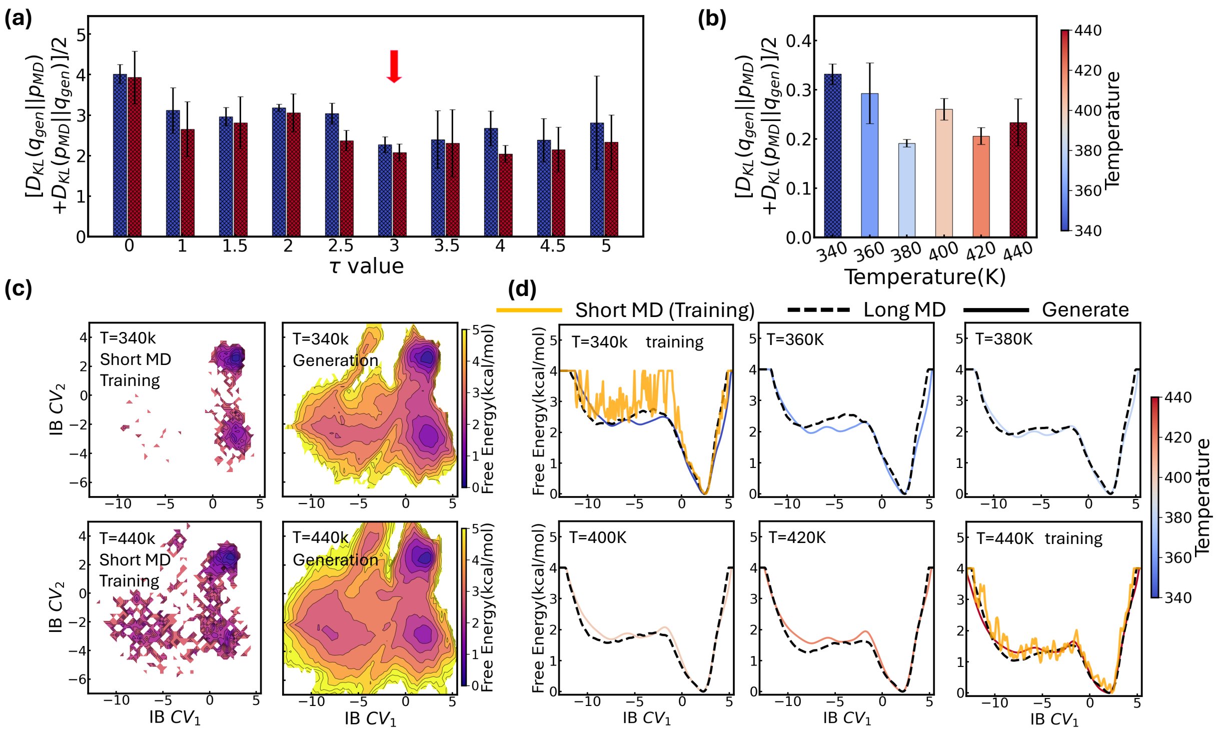

Figure S6: Benchmarking the generative performance of Latent Thermodynamic Flows (LaTF) trained with highly limited data. The LaTF model is trained using the first $1\mu s$ segment of Chignolin protein MD trajectory collected at 340 K and 440 K. (a) Symmetric Kullback–Leibler (KL) divergence between the latent-space distributions of encoded $1\mu s$ MD trajectories and LaTF-generated samples with different prior tilting parameters at the two training temperatures, 340 K and 440 K. The optimal tilting factor is determined to be $\tau = 3$ (indicated by the red arrow), which results in the lowest KL divergence across the training temperatures. (b) Symmetric KL divergence between the latent-space distributions generated by LaTF and the reference distributions encoded from full ultra-long MD trajectories across various temperatures. All uncertainties are estimated by training the LaTF model ten times with different random initialization seeds. (c) Free energy landscapes in the latent space estimated from $1\mu s$ training MD trajectories (first column) and from latent samples generated by LaTF models trained on limited data (second column). (d) Comparison of free energy landscapes obtained from $1\mu s$ short MD trajectories (orange solid), ultra-long MD trajectories (black dashed), and LaTF-generated samples (colorful solid).

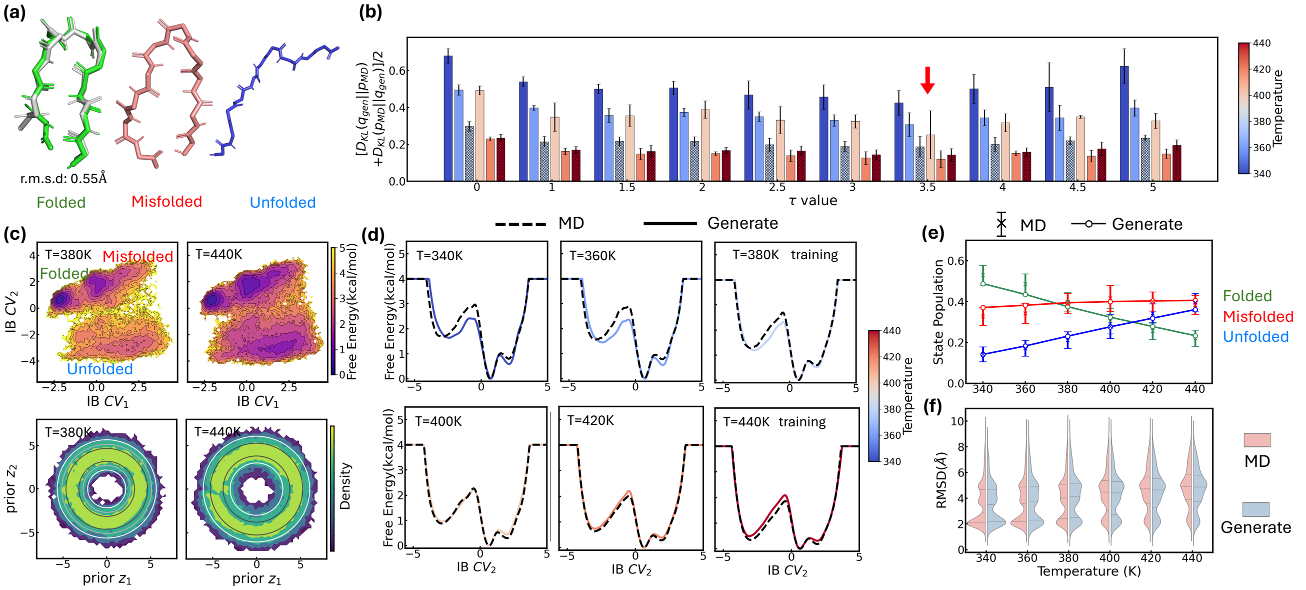

Figure S7: Benchmarking the generative capability of Latent Thermodynamic Flows (LaTF) in low-temperature regimes when trained on data from comparatively higher temperatures for Chignolin protein. The LaTF model is trained using data collected at 380 K and 440 K, and its generative performance is evaluated across other temperatures. The MD trajectory at each training temperature is uniformly divided into five segments, with four segments used for training and one for validation. (a) Backbone structures of the highest-probability conformations scored by LaTF ($\tau = 3.5$) for the Chignolin protein, corresponding to different states. The highest-scoring folded structure (green) is overlaid with the NMR structure (grey), and the backbone RMSD is computed. (b) Symmetric Kullback–Leibler (KL) divergence between the LaTF-generated distribution and the encoded MD distribution in the IB latent space across different temperatures. Training temperatures are indicated by shaded colors. KL divergences are computed using only the validation set at training temperatures and the full dataset at other generative temperatures. The error bars are quantified accordingly using the cross-validation results. The optimal tilting factor, $\tau = 3.5$, is selected based on KL divergence evaluated on the validation dataset at the training temperatures and is subsequently used to generate all other results (highlighted by the red arrow). (c) Free energy landscapes estimated from the encoded MD data in latent space are shown for 380 K and 440 K, with the corresponding states indicated (top row). The encoded MD data are then transformed forward to the prior distribution, and the resulting distributions are visualized (bottom row). Contour lines derived from the analytical prior are overlaid to assess the performance of the normalizing flow in mapping between the distributions. (d) Comparison between the generative energy landscapes produced by LaTF (solid lines) and the reference obtained from ultra-long MD simulations (dashed lines) across six temperatures. (e) Populations of the three states predicted by LaTF models trained at 380 K and 440 K across a range of temperatures. Error bars indicate state populations estimated from Markov State Models (MSMs) constructed using ultra-long MD trajectories at each temperature, with state assignments based on the trained LaTF. Uncertainties are computed using a Bayesian approach[65]. (f) Comparison of RMSD distributions relative to the NMR structure for MD data and LaTF-generated samples. LaTF samples are reconstructed into all-atom structures using their nearest neighbors in the latent IB space, which are encoded from the training MD data. Distribution quartiles are marked with dashed lines.

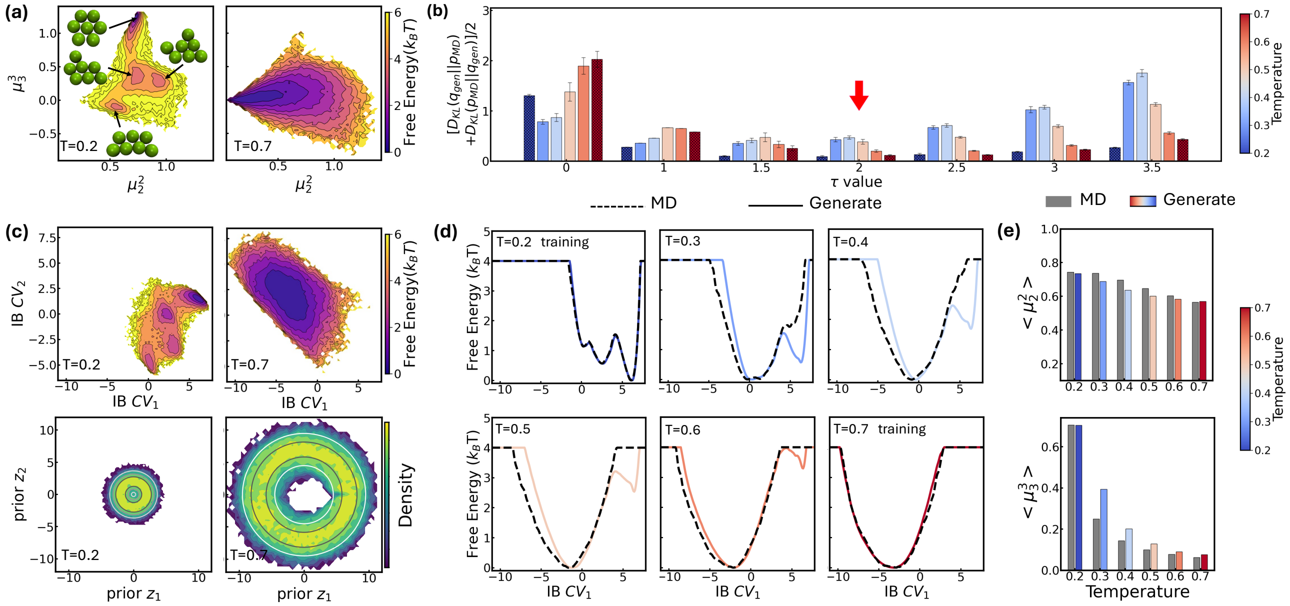

Figure S8: Benchmarking the generative performance of Latent Thermodynamic Flows (LaTF) trained on datasets acquired at two widely separated temperatures for the Lennard-Jones 7 (LJ7) system. The LaTF model is trained on data collected at $0.2\epsilon/k_B$ and $0.7\epsilon/k_B$, and used to generate samples at four intermediate temperatures. Long trajectories collected at $0.2\epsilon/k_B$ and $0.7\epsilon/k_B$ are evenly partitioned into five segments, with four segments used for training and the remaining one used for validation. (a) Free energy landscapes projected onto two physical order parameters, i.e., the second and third moments of coordination numbers (μ_2^2 and μ_3^3)[32], estimated from long MD simulations at the two training temperatures. For $0.2\epsilon/k_B$, representative structures corresponding to the highest probability conformations within each metastable state, as scored by LaTF, are also visualized. (b) Symmetric Kullback–Leibler (KL) divergence between the latent distributions generated by LaTF and the ground-truth distributions encoded directly from MD data. For the two training temperatures, KL divergences are computed only with respect to the validation datasets and highlighted with shading. For all other temperatures, KL divergences are evaluated against the full MD datasets. The optimal prior tilting factor is identified as $\tau = 2$, as it yields the lowest KL divergence at the training temperatures (highlighted by red arrow). (c) Free energy landscapes projected onto the latent IB space using full MD data for the two training temperatures (top row), and corresponding distributions of forward-flowed encoded MD data in the target prior space (bottom row). Contour lines represent the analytical prior and are used to assess LaTF's ability to learn an effective and invertible transformation between the latent and prior distributions. (d) Comparison between the generative free energy landscape (solid line) and the reference free energy landscape obtained from long MD trajectories (dashed line) across six different temperatures. (e) Comparison of the generative (colored bars) and reference (gray bars) means of the second and third moments of coordination numbers across different temperatures. Generative samples are reconstructed into full-coordinate structures by replacing each with its nearest neighbor in the latent IB space.

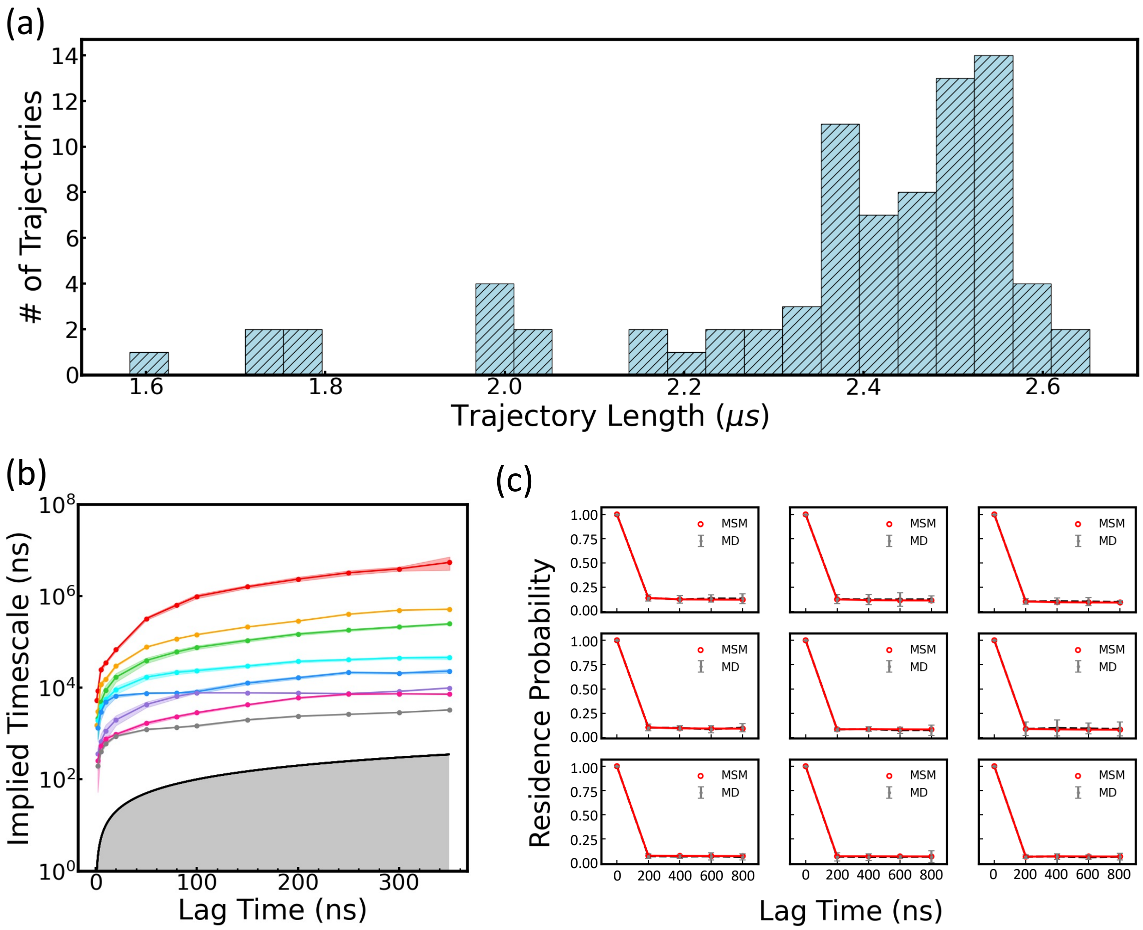

Figure S9: Construction and validation of the 200-microstate Markov State Model (MSM) using Implied Timescale (ITS) analysis and the Chapman-Kolmogorov (CK) test for GCAA tetraloop RNA at 300 K. (a) Distribution of simulated MD trajectory lengths for the GCAA tetraloop at 300 K. A total of 80 trajectories were run in parallel, yielding an aggregate simulation time of $\sim 189\mu s$ and an average trajectory length of $\sim 2.37\mu s$. (b) ITS calculated using models constructed with varying lag times. The ITS curves converge beyond a lag time of 200 ns, indicating that models exhibit Markovian behavior with lag times greater than 200 ns. (c) CK test of the MSM constructed with a 200 ns lag time for the nine most populated microstates. The agreement between residence probabilities derived from raw MD data and those predicted by the MSM supports the model's accuracy in capturing long-timescale dynamics. Uncertainties in the ITS and CK test error bars are estimated by bootstrapping the trajectory ensemble 50 times with replacement.

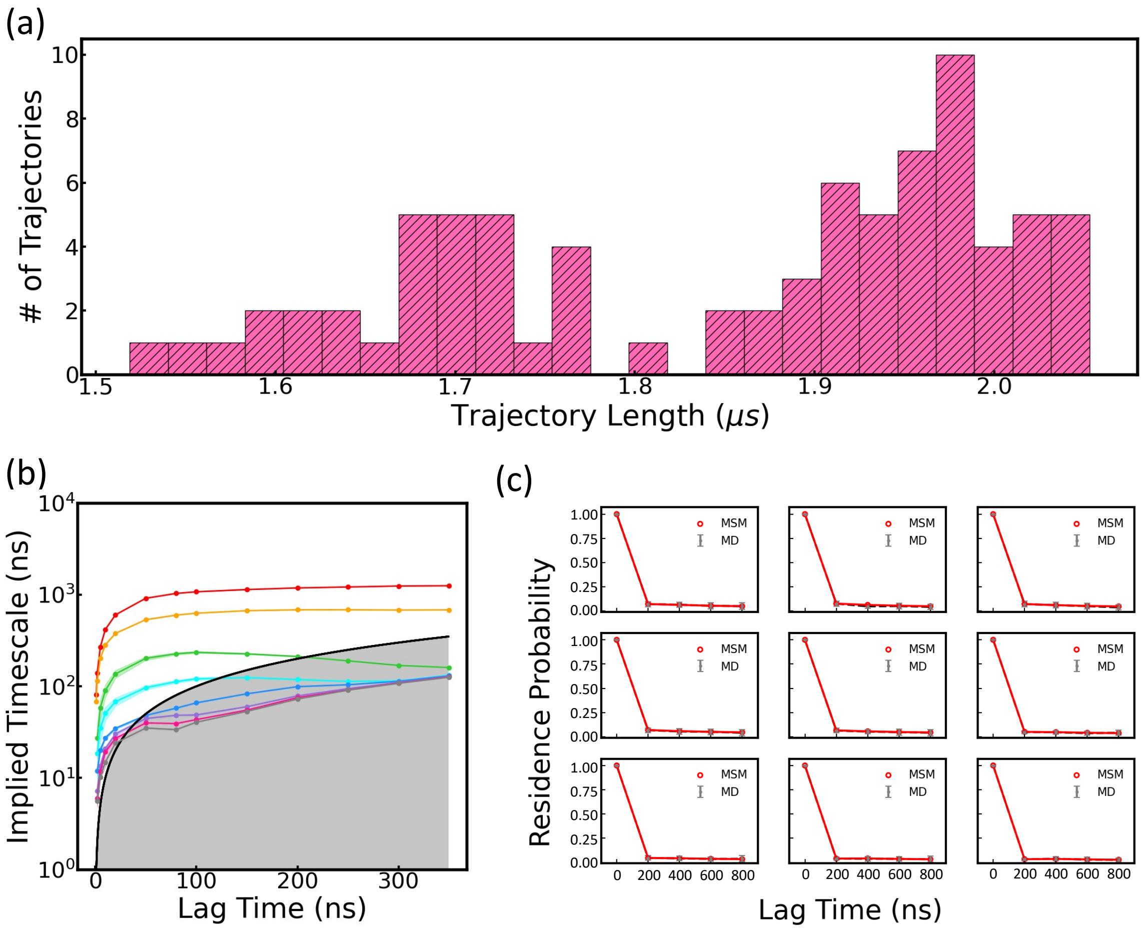

Figure S10: Construction and validation of the 200-microstate Markov State Model (MSM) using Implied Timescale (ITS) analysis and the Chapman-Kolmogorov (CK) test for GCAA tetraloop RNA at 400 K. (a) Distribution of simulated MD trajectory lengths for the GCAA tetraloop at 400 K. A total of 80 trajectories were run in parallel, yielding an aggregate simulation time of $\sim 148\mu s$ and an average trajectory length of $\sim 1.85\mu s$. (b) ITS calculated using models constructed with varying lag times. The ITS curves converge beyond a lag time of 200 ns, indicating that models exhibit Markovian behavior with lag times greater than 200 ns. (c) CK test of the MSM constructed with a 200 ns lag time for the nine most populated microstates. The agreement between residence probabilities derived from raw MD data and those predicted by the MSM supports the model's accuracy in capturing long-timescale dynamics. Uncertainties in the ITS and CK test error bars are estimated by bootstrapping the trajectory ensemble 50 times with replacement.

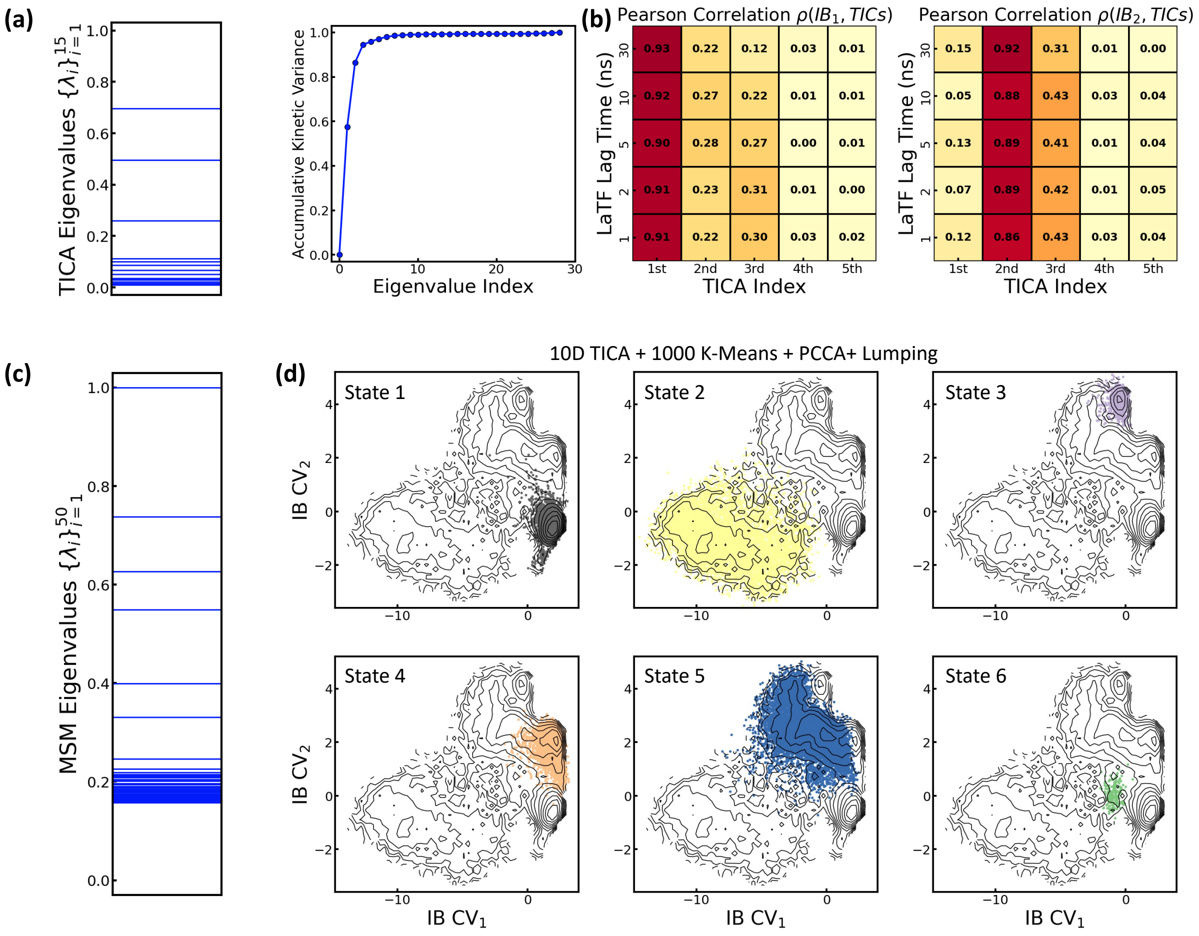

Figure S11: Evaluation of the capacity of Latent Thermodynamic Flows (LaTF) to represent and separate metastable states within the two-dimensional information bottleneck (IB) space for the Chignolin folding system at T = 340 K (a) The left panel shows the eigenvalue spectrum of the covariance matrix–whitened time-lagged correlation matrix at lag time of 30 ns, computed using C_α pairwise distances as input features. The right panel shows the cumulative sum of the ranked tICA eigenvalues, highlighting the contributions of the leading components. (b) Pearson correlations between the IB coordinates learned by LaTF models trained with different lag times and the time-lagged independent components obtained from tICA at a lag time of 30 ns. (c) Eigenvalue spectrum of the transition probability matrix (TPM) for the Markov State Model (MSM) constructed at a 30 ns lag time. The MSM is built from 1,000 microstates obtained via K-means clustering in the space spanned by the first twenty time-lagged independent components derived from tICA. (d) Visualization of the six metastable states identified by the MSM in the two-dimensional IB space. The states were obtained by lumping the 1,000 microstates using the Perron–cluster cluster analysis plus (PCCA+) algorithm. Each protein conformation is assigned a metastable-state label and shown in the IB space with a distinct color.

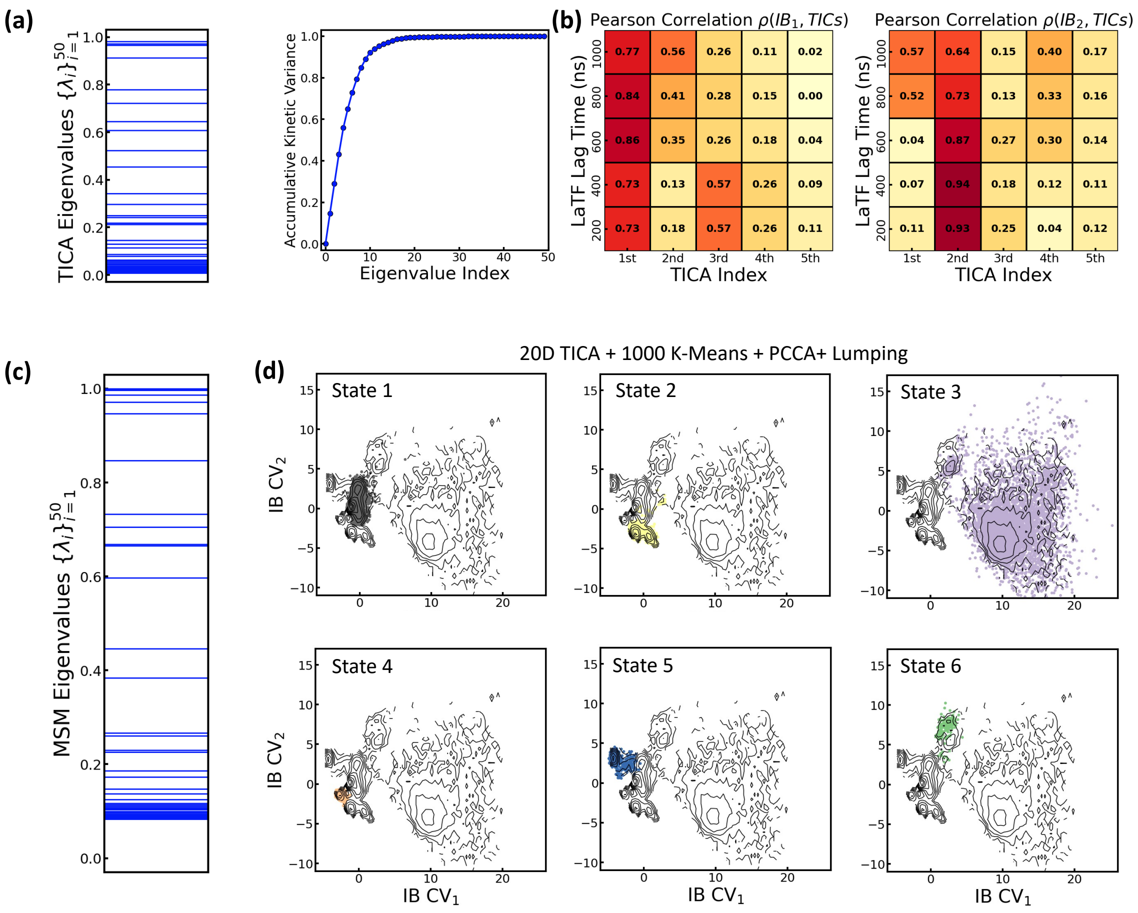

Figure S12: Evaluation of the capacity of Latent Thermodynamic Flows (LaTF) to represent and separate metastable states within the two-dimensional information bottleneck (IB) space for the RNA Tetraloop folding system at T = 300 K (a) The left panel shows the eigenvalue spectrum of the covariance matrix–whitened time-lagged correlation matrix at lag time of 200 ns, computed using **r**-vector and and selected pairwise carbon distances as input features. The right panel shows the cumulative sum of the ranked tICA eigenvalues, highlighting the contributions of the leading components. (b) Pearson correlations between the IB coordinates learned by LaTF models trained with different lag times and the time-lagged independent components obtained from tICA at a lag time of 200 ns. (c) Eigenvalue spectrum of the TPM for the MSM constructed at a 200 ns lag time. The MSM is built from 1,000 microstates obtained via K-means clustering in the space spanned by the first ten time-lagged independent components derived from tICA. (d) Visualization of the six metastable states identified by the MSM in the two-dimensional IB space. The states were obtained by lumping the 1,000 microstates using the PCCA+ algorithm. Each RNA conformation is assigned a metastable-state label and shown in the IB space with a distinct color.

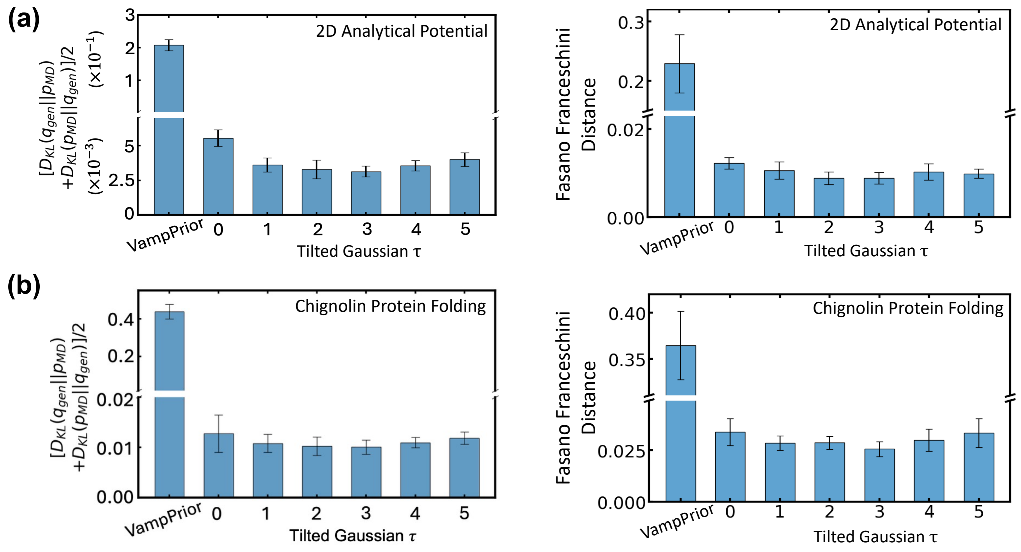

Figure S13: Comparison of the Kullback–Leibler (KL) divergence and the Fasano–Franceschini test in the quantification of distributional differences. For (a) the 2D analytical potential and (b) Chignolin folding at 340 K, the symmetric KL divergence and Fasano–Franceschini distance between the reference IB distribution (derived from the validation dataset) and the distributions generated by vanilla SPIB with VampPrior and LaTF models under varying tilting factors are computed and shown. Uncertainties are estimated using five-fold cross-validation.

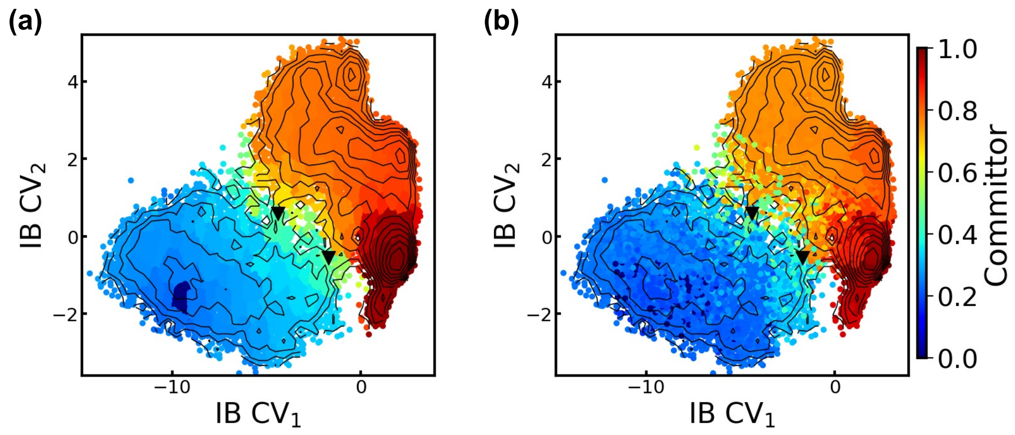

Figure S14: Visualization of the committor function distribution for Chignolin protein folding at T = 340 K in the LaTF two-dimensional IB space. (a) Committor function distribution quantified using a 200-microstate MSM constructed in the IB space. (b) Committor function distribution evaluated using another independent MSM with 1000 microstates built in the ten-dimensional tICA space. In both cases, projected conformations in the IB space are colored based on their corresponding microstate committor values. Saddle points are identified using kernel density estimation and denoted by black inverted triangles.

[1] Dedi Wang and Pratyush Tiwary. State predictive information bottleneck. *The Journal of Chemical Physics*, 154(13), 2021.

[2] Dedi Wang, Yunrui Qiu, Eric R Beyerle, Xuhui Huang, and Pratyush Tiwary. Information bottleneck approach for markov model construction. *Journal of chemical theory and computation*, 20(12):5352–5367, 2024.

[3] Suemin Lee, Dedi Wang, Markus A Seeliger, and Pratyush Tiwary. Calculating protein–ligand residence times through state predictive information bottleneck based enhanced sampling. *Journal of Chemical Theory and Computation*, 20(14):6341–6349, 2024.

[4] Bodhi P Vani, Akashnathan Aranganathan, Dedi Wang, and Pratyush Tiwary. Alphafold2-rave: From sequence to boltzmann ranking. *Journal of chemical theory and computation*, 19(14):4351–4354, 2023.

[5] Naftali Tishby, Fernando C Pereira, and William Bialek. The information bottleneck method. *arXiv preprint physics/0004057*, 2000.

[6] Alexander A Alemi, Ian Fischer, Joshua V Dillon, and Kevin Murphy. Deep variational information bottleneck. *arXiv preprint arXiv:1612.00410*, 2016.

[7] Diederik P Kingma, Max Welling, et al. Auto-encoding variational bayes, 2013.

[8] Jianlin Su and Guang Wu. f-vaes: Improve vaes with conditional flows. *arXiv preprint arXiv:1809.05861*, 2018.

[9] Arash Vahdat, Karsten Kreis, and Jan Kautz. Score-based generative modeling in latent space. *Advances in neural information processing systems*, 34:11287–11302, 2021.

[10] Quan Dao, Hao Phung, Binh Nguyen, and Anh Tran. Flow matching in latent space. *arXiv preprint arXiv:2307.08698*, 2023.

[11] Griffin Floto, Stefan Kremer, and Mihai Nica. The tilted variational autoencoder: Improving out-of-distribution detection. In *The Eleventh International Conference on Learning Representations*, 2023.

[12] Samuel Tamagnone, Alessandro Laio, and Marylou Gabrié. Coarse-grained molecular dynamics with normalizing flows. *Journal of Chemical Theory and Computation*, 20(18):7796–7805, 2024.

[13] Frank Noé, Simon Olsson, Jonas Köhler, and Hao Wu. Boltzmann generators: Sampling equilibrium states of many-body systems with deep learning. *Science*, 365(6457):eaaw1147, 2019.

[14] Lukas Herron, Kinjal Mondal, John S Schneekloth Jr, and Pratyush Tiwary. Inferring phase transitions and critical exponents from limited observations with thermodynamic maps. *Proceedings of the National Academy of Sciences*, 121(52):e2321971121, 2024.

[15] Yihang Wang, Lukas Herron, and Pratyush Tiwary. From data to noise to data for mixing physics across temperatures with generative artificial intelligence. *Proceedings of the National Academy of Sciences*, 119(32):e2203656119, 2022.

[16] Laurent Dinh, Jascha Sohl-Dickstein, and Samy Bengio. Density estimation using real nvp. *arXiv preprint arXiv:1605.08803*, 2016.

[17] George Papamakarios, Theo Pavlakou, and Iain Murray. Masked autoregressive flow for density estimation. *Advances in neural information processing systems*, 30, 2017.

[18] Hao Wu, Jonas Köhler, and Frank Noé. Stochastic normalizing flows. *Advances in neural information processing systems*, 33:5933–5944, 2020.

[19] Durk P Kingma and Prafulla Dhariwal. Glow: Generative flow with invertible 1x1 convolutions. *Advances in neural information processing systems*, 31, 2018.

[20] Conor Durkan, Artur Bekasov, Iain Murray, and George Papamakarios. Neural spline flows. *Advances in neural information processing systems*, 32, 2019.

[21] Benedict Leimkuhler and Charles Matthews. Efficient molecular dynamics using geodesic integration and solvent–solute splitting. *Proceedings of the Royal Society A: Mathematical, Physical and Engineering Sciences*, 472(2189):20160138, 2016.

[22] Shinya Honda, Kazuhiko Yamasaki, Yoshito Sawada, and Hisayuki Morii. 10 residue folded peptide designed by segment statistics. *Structure*, 12(8):1507–1518, 2004.

[23] Pekka Mark and Lennart Nilsson. Structure and dynamics of the tip3p, spc, and spc/e water models at 298 k. *The Journal of Physical Chemistry A*, 105(43):9954–9960, 2001.

[24] William L Jorgensen, David S Maxwell, and Julian Tirado-Rives. Development and testing of the opls all-atom force field on conformational energetics and properties of organic liquids. *Journal of the american chemical society*, 118(45):11225–11236, 1996.

[25] Michael J Robertson, Julian Tirado-Rives, and William L Jorgensen. Improved peptide and protein torsional energetics with the opls-aa force field. *Journal of chemical theory and computation*, 11(7):3499–3509, 2015.

[26] Tim Marshall, Robert Raddi, and Vincent Voelz. An evaluation of force field accuracy for the mini-protein chignolin using markov state models. *Biophysical Journal*, 122(3):420a, 2023.

[27] Tom Darden, Darrin York, Lee Pedersen, et al. Particle mesh ewald: An n log (n) method for ewald sums in large systems. *Journal of chemical physics*, 98:10089–10089, 1993.

[28] Berk Hess, Henk Bekker, Herman JC Berendsen, and Johannes GEM Fraaije. Lincs: A linear constraint solver for molecular simulations. *Journal of computational chemistry*, 18(12):1463–1472, 1997.

[29] Herman JC Berendsen, JPM van Postma, Wilfred F Van Gunsteren, ARHJ DiNola, and Jan R Haak. Molecular dynamics with coupling to an external bath. *The Journal of chemical physics*, 81(8):3684–3690, 1984.

[30] M Parrinello and A Rahman. Strain fluctuations and elastic constants. *The Journal of Chemical Physics*, 76(5):2662–2666, 1982.

[31] Mark James Abraham, Teemu Murtola, Roland Schulz, Szilárd Páll, Jeremy C Smith, Berk Hess, and Erik Lindahl. Gromacs: High performance molecular simulations through multi-level parallelism from laptops to supercomputers. *SoftwareX*, 1:19–25, 2015.

[32] Gareth A Tribello, Michele Ceriotti, and Michele Parrinello. A self-learning algorithm for biased molecular dynamics. *Proceedings of the National Academy of Sciences*, 107(41):17509–17514, 2010.

[33] Peter Schwerdtfeger and David J Wales. 100 years of the lennard-jones potential. *Journal of Chemical Theory and Computation*, 20(9):3379–3405, 2024.

[34] Ziyue Zou, Dedi Wang, and Pratyush Tiwary. A graph neural network-state predictive information bottleneck (gnn-spib) approach for learning molecular thermodynamics and kinetics. *Digital Discovery*, 4(1):211–221, 2025.

[35] Gareth A Tribello, Massimiliano Bonomi, Davide Branduardi, Carlo Camilloni, and Giovanni Bussi. Plumed 2: New feathers for an old bird. *Computer physics communications*, 185(2):604–613, 2014.

[36] Ivo L Hofacker, Walter Fontana, Peter F Stadler, L Sebastian Bonhoeffer, Manfred Tacker, Peter Schuster, et al. Fast folding and comparison of rna secondary structures. *Monatshefte fur chemie*, 125:167–167, 1994.

[37] Ronny Lorenz, Stephan H Bernhart, Christian Höner zu Siederdissen, Hakim Tafer, Christoph Flamm, Peter F Stadler, and Ivo L Hofacker. Viennarna package 2.0. *Algorithms for molecular biology*, 6:1–14, 2011.

[38] Rhiju Das and David Baker. Macromolecular modeling with rosetta. *Annu. Rev. Biochem.*, 77(1):363–382, 2008.

[39] Rhiju Das, John Karanicolas, and David Baker. Atomic accuracy in predicting and designing noncanonical rna structure. *Nature methods*, 7(4):291–294, 2010.

[40] Andrew Martin Watkins, Ramya Rangan, and Rhiju Das. Farfar2: improved de novo rosetta prediction of complex global rna folds. *Structure*, 28(8):963–976, 2020.

[41] Sandro Bottaro, Francesco Di Palma, and Giovanni Bussi. The role of nucleobase interactions in rna structure and dynamics. *Nucleic acids research*, 42(21):13306–13314, 2014.

[42] Dazhi Tan, Stefano Piana, Robert M Dirks, and David E Shaw. Rna force field with accuracy comparable to state-of-the-art protein force fields. *Proceedings of the National Academy of Sciences*, 115(7):E1346–E1355, 2018.

[43] James A Maier, Carmenza Martinez, Koushik Kasavajhala, Lauren Wickstrom, Kevin E Hauser, and Carlos Simmerling. ff14sb: improving the accuracy of protein side chain and backbone parameters from ff99sb. *Journal of chemical theory and computation*, 11(8):3696–3713, 2015.

[44] Maxwell R Tucker, Stefano Piana, Dazhi Tan, Michael V LeVine, and David E Shaw. Development of force field parameters for the simulation of single-and double-stranded dna molecules and dna–protein complexes. *The Journal of Physical Chemistry B*, 126(24):4442–4457, 2022.

[45] Jose LF Abascal and Carlos Vega. A general purpose model for the condensed phases of water: Tip4p/2005. *The Journal of chemical physics*, 123(23), 2005.

[46] Alex D MacKerell Jr, Donald Bashford, MLDR Bellott, Roland Leslie Dunbrack Jr, Jeffrey D Evanseck, Martin J Field, Stefan Fischer, Jiali Gao, Houyang Guo, Sookhee Ha, et al. All-atom empirical potential for molecular modeling and dynamics studies of proteins. *The journal of physical chemistry B*, 102(18):3586–3616, 1998.

[47] Yusuke Naritomi and Sotaro Fuchigami. Slow dynamics in protein fluctuations revealed by time-structure based independent component analysis: the case of domain motions. *The Journal of chemical physics*, 134(6), 2011.

[48] Frank Noé and Cecilia Clementi. Kinetic distance and kinetic maps from molecular dynamics simulation. *Journal of chemical theory and computation*, 11(10):5002–5011, 2015.

[49] Robert T McGibbon and Vijay S Pande. Variational cross-validation of slow dynamical modes in molecular kinetics. *The Journal of chemical physics*, 142(12), 2015.

[50] Hao Wu and Frank Noé. Variational approach for learning markov processes from time series data. *Journal of Nonlinear Science*, 30(1):23–66, 2020.

[51] Jan-Hendrik Prinz, Hao Wu, Marco Sarich, Bettina Keller, Martin Senne, Martin Held, John D Chodera, Christof Schütte, and Frank Noé. Markov models of molecular kinetics: Generation and validation. *The Journal of chemical physics*, 134(17), 2011.

[52] Sandro Bottaro, Giovanni Bussi, Giovanni Pinamonti, Sabine Reißer, Wouter Boomsma, and Kresten Lindorff-Larsen. Barnaba: software for analysis of nucleic acid structures and trajectories. *RNA*, 25(2):219–231, 2019.

[53] Guillermo Pérez-Hernández, Fabian Paul, Toni Giorgino, Gianni De Fabritiis, and Frank Noé. Identification of slow molecular order parameters for markov model construction. *The Journal of chemical physics*, 139(1), 2013.

[54] Frank Noé, Illia Horenko, Christof Schütte, and Jeremy C Smith. Hierarchical analysis of conformational dynamics in biomolecules: Transition networks of metastable states. *Journal of Chemical Physics*, 126(15), 2007.

[55] Shams Mehdi, Zachary Smith, Lukas Herron, Ziyue Zou, and Pratyush Tiwary. Enhanced sampling with machine learning. *Annual Review of Physical Chemistry*, 75(2024):347–370, 2024.

[56] Vanessa J Meraz, Ziyue Zou, and Pratyush Tiwary. Simulating crystallization in a colloidal system using state predictive information bottleneck based enhanced sampling. *The Journal of Physical Chemistry B*, 128(34):8207–8214, 2024.

[57] Ruiyu Wang, Shams Mehdi, Ziyue Zou, and Pratyush Tiwary. Is the local ion density sufficient to drive nacl nucleation from the melt and aqueous solution? *The Journal of Physical Chemistry B*, 128(4):1012–1021, 2024.

[58] Dedi Wang and Pratyush Tiwary. Augmenting human expertise in weighted ensemble simulations through deep learning-based information bottleneck. *Journal of Chemical Theory and Computation*, 20(23):10371–10383, 2024.

[59] Shams Mehdi, Dedi Wang, Shashank Pant, and Pratyush Tiwary. Accelerating all-atom simulations and gaining mechanistic understanding of biophysical systems through state predictive information bottleneck. *Journal of Chemical Theory and Computation*, 18(5):3231–3238, 2022.

[60] Mingyuan Zhang, Zhicheng Zhang, Hao Wu, and Yong Wang. Flow matching for optimal reaction coordinates of biomolecular systems. *Journal of Chemical Theory and Computation*, 21(1):399–412, 2024.

[61] Alex Rodriguez and Alessandro Laio. Clustering by fast search and find of density peaks. *Science*, 344(6191):1492–1496, 2014.

[62] Ken Shoemake. Animating rotation with quaternion curves. In *Proceedings of the 12th annual conference on Computer graphics and interactive techniques*, pages 245–254, 1985.

[63] Eric Vanden-Eijnden. Transition path theory. In *Computer Simulations in Condensed Matter Systems: From Materials to Chemical Biology Volume 1*, pages 453–493. Springer, 2006.

[64] Frank Noé, Christof Schütte, Eric Vanden-Eijnden, Lothar Reich, and Thomas R Weikl. Constructing the equilibrium ensemble of folding pathways from short off-equilibrium simulations. *Proceedings of the National Academy of Sciences*, 106(45):19011–19016, 2009.

[65] Philipp Metzner, Frank Noé, and Christof Schütte. Estimating the sampling error: Distribution of transition matrices and functions of transition matrices for given trajectory data. *Physical Review E—Statistical, Nonlinear, and Soft Matter Physics*, 80(2):021106, 2009.
